# Opportunities to integrate herders’ indicators into formal rangeland monitoring: an example from Mongolia

**DOI:** 10.1002/eap.1899

**Published:** 2019-05-17

**Authors:** Chantsallkham Jamsranjav, María E. Fernández‐Giménez, Robin S. Reid, B. Adya

**Affiliations:** ^1^ Department of Forest and Rangeland Stewardship Colorado State University Fort Collins Colorado 80523‐1472 USA; ^2^ Center for Collaborative Conservation Colorado State University Fort Collins Colorado 80523‐1472 USA; ^3^ Department of Ecosystem Science and Sustainability Colorado State University Fort Collins Colorado 80523‐1472 USA; ^4^ Nutag Action Research Institute Ulaanbaatar Mongolia

**Keywords:** community‐based monitoring, community‐based rangeland management, ecological indicators, indigenous knowledge, integrated indicators, local knowledge, Mongolia, participatory monitoring, pastoralist, rangeland condition, rangeland monitoring, traditional ecological knowledge

## Abstract

Despite increasing calls for knowledge integration around the world, traditional knowledge is rarely used in formal, Western‐science‐based monitoring and resource management. To better understand indicators herders use and their relationship to researcher‐measured indicators, we conducted in‐depth field interviews with 26 herders in three ecological zones of Mongolia. We asked each herder to (1) assess the overall condition of three different sites located along a livestock‐use gradient from their winter camp using a numeric scale, (2) describe the indicators they used in their assessment, and (3) explain what caused their pastures to remain healthy or become degraded. At each site, we collected field data on vegetation variables and compared these with herders’ ratings and indicators using linear regression. We used classification and ordination to understand how herders’ assessment scores related to plant community composition, and determine how well multivariate analysis of factors determining plant community composition aligned with herders’ observations of factors causing rangeland change. Across all ecological zones, herders use indicators similar to those used in formal monitoring. Herders’ assessment scores correlated significantly and positively with measured total foliar cover in all three ecological zones, and with additional measured variables in the steppe and desert steppe. Ordination revealed that herder assessment scores were correlated with the primary ordination axis in each zone, and the main factors driving plant community composition in each zone were the same as those identified by herders as the primary causes of rangeland change in that zone. These results show promise for developing integrated indicators and monitoring protocols and highlight the importance of developing a common language of monitoring terminology shared by herders, government monitoring agencies, and researchers. We propose a new model for integrating herder knowledge and participation into formal monitoring in Mongolia, with implications for rangelands and pastoral people globally. We suggest practical ways of involving herders in formal monitoring that have potential broad application for promoting local and indigenous people's participation in implementing international agreements such as the UN Convention to Combat Desertification and the UN Convention on Biological Diversity, both of which call for involvement of local people and indigenous/traditional knowledges.

## Introduction

Both international environmental agreements (e.g., UN Convention to Combat Desertification, UN Convention on Biodiversity) and national environmental management agencies (e.g., U.S. Fish and Wildlife Service), increasingly recognize the value of local people's participation and specifically, of incorporating indigenous, traditional, and local knowledges, to achieve effective, just, and equitable implementation. As rangelands account for at least 40% of Earth's land area (Asner et al. 2004, Briske 2017), and their sustainable management is essential for combatting desertification and maintaining biodiversity, the participation of pastoral people that live in these areas, and incorporation of their knowledge in monitoring and management, is especially critical.

Traditional ecological knowledge (TEK) is a vibrant knowledge system held by geographically and socially defined communities in relation to their day‐to‐day interactions and relationships with their environment (Berkes 1999, Fernandez‐Gimenez and Fillat 2012). Traditional ecological knowledge is evolving knowledge that is accumulated, practiced and transmitted from one generation to another, primarily through observation and imitation (Berkes et al. 2000, Tang and Gavin 2010, Fernandez‐Gimenez and Fillat 2012). Traditional ecological knowledge also represents a place‐based value and belief system (Berkes et al. 2000, Raymond et al. 2010), and is reflected in management institutions, such as harvest prohibitions, sacred groves, etc. (Agrawal and Gibson 1999, Berkes 2009, Samakov and Berkes 2017). TEK is similar to indigenous knowledge (IK), culturally embedded knowledge of indigenous groups (Snively and Corsiglia 2001, Gadgil et al. 2003), and local knowledge (LK, experiential knowledge of local people; Olsson and Folke 2001, Raymond et al. 2010). TEK studies have been conducted in many ecosystems around the world, including forests, grasslands, and marine systems (Fernandez‐Gimenez 2000, Stave et al. 2007, Parlee et al. 2012, Narchi et al. 2014, Molnar 2017). In rangelands, researchers have documented pastoralist knowledge in many different regions, including Asia (Fernandez‐Gimenez 2000, Tang and Gavin 2010, Bruegger et al. 2014, Kakinuma et al. 2014, Hopping et al. 2016, Yeh et al. 2017), Europe (Fernandez‐Gimenez and Fillat 2012, Molnar 2017), Africa (Mapinduzi et al. 2003, Reed et al. 2007, 2013, Stringer and Reed 2007, Roba and Oba 2009, Raymond et al. 2010, Jandreau and Berkes 2016), the USA (Knapp and Fernandez‐Gimenez 2008), and Australia (Moller et al. 2004, Waudby et al. 2013). These studies indicate that pastoralists’ assessments of rangelands focus on plant community composition and plant growth (Fernandez‐Gimenez 2000, Kakinuma et al. 2014, Jandreau and Berkes 2016), nutritional value for livestock (Mapinduzi et al. 2003, Roba and Oba 2009, Fernandez‐Gimenez and Fillat 2012, Bruegger et al. 2014), soil‐related changes (Stringer and Reed 2007, Bruegger et al. 2014), livestock condition and production (Roba and Oba 2009, Fernandez‐Gimenez and Fillat 2012, Hopping et al. 2016), and animal behavior in relation to rangeland forage quality (Molnar 2017).

Both Western scientific and traditional knowledges have limitations but combining these knowledge types may help overcome them. Western scientific knowledge is often decontextualized and systematic methods of assessing degradation may miss context specific information embedded in local knowledges (Stringer and Reed 2007, Stringer et al. 2014). Hereafter, we use “science” and “scientific” to refer to Western science. We recognize the existence of indigenous science as a distinct and valid process of generating knowledge (Snively and Corsiglia 2001, Raymond et al. 2010, Tengӧ et al. 2014), but use “science” as shorthand for “Western science” for simplicity. In addition, scientific monitoring data are often sparse in their spatial and temporal coverage of vast rangelands and long time periods. Formal monitoring and scientific data are frequently collected at scales that are too fine (i.e., a few plot‐based monitoring points to characterize a large landscape), too coarse (i.e., remotely sensed data that lacks sufficient detail or resolution), or too infrequent to guide management decisions (Stringer and Reed 2007, Reed et al. 2008, Klein et al. 2014). TEK observations of land condition changes are generally not systematic or documented, and interpretation of these observations is shaped by observers’ different uses and values: what is degraded for one person may be valuable for another (Reed et al. 2013). Both scientific knowledge and TEK contain uncertainties, assumptions, and value judgements, and both develop through processes of investigation, observation, and experience (Clark and Murdoch 1997). However, these similarities and the potential complementarities of scientific knowledge and TEK are often overlooked by researchers and herders alike, each of whom may view the other's knowledge with suspicion and treat it as lacking credibility or relevance. Science often is not understood by, credible, or useful to non‐scientists. This is because scientific studies often have little relevance to local decision makers, are not context specific, problem solving, or outcome oriented, or fail to examine the interactions between social, ecological, and economic phenomena and instead focus more on outputs, articles, methods, and trainings (Kristjanson et al. 2009). Similarly, TEK often is not understood by, credible, or useful to scientists, because TEK is locally specific and sometimes challenging to relate to larger‐scale changes such as climate change (Cash et al. 2006).

Despite these potential challenges, recent attempts to address rangeland degradation and promote community participation in rangeland management call for integrating herders’ knowledge with knowledge held by scientists (Roba and Oba 2009, Reed et al. 2013, 2014). This combined understanding could support development of integrated indicators and monitoring protocols that are meaningful, credible, and useful to herders, managers and scientists. This knowledge integration process and the resulting integrated indicators and monitoring protocols could lay the groundwork for successful implementation of participatory monitoring, and hence for increased accuracy, coverage, and relevance of land degradation assessments (Stringer and Reed 2007).

Participatory monitoring involves diverse stakeholders with different interests and different types and levels of knowledge and experiences in designing and conducting monitoring (Danielsen et al. 2005, Fernandez‐Gimenez et al. 2008, Singh et al. 2014). Participatory community‐based monitoring has several potential benefits. For example, community engagement and participation in resource monitoring can build trust internally and credibility externally (Fernandez‐Gimenez et al. 2008), increase the likelihood that monitoring data will be used to make decisions, and lead to faster action based on monitoring outcomes (Brook et al. 2009, Danielsen et al. 2014, Johnson et al. 2015). Local people's participation in formal data collection and monitoring can enhance local capacity, including the ability to detect changes and influence management. For example, local people may increase spatial coverage of monitoring because they spend time on and observe more and larger areas than a few formal monitoring points. When local people participate in formal monitoring, especially when monitoring indicators are informed by local knowledge and co‐developed with researchers, monitoring has the potential for greater spatial coverage (more observation points) and more frequent observations by people who are out on the land daily (Herrick et al. 2010). In South Africa, flexible, adaptive, and easy to use local monitoring protocols have been developed to improve the relevance of land degradation assessment (Kellner and Moussa 2009). If the monitoring methods and data collection and recording protocols are simple and locally appropriate, then co‐designed, community‐based, monitoring methods can be sustained at the local level (Danielsen et al. 2005). Making the monitoring indicators meaningful and feasible to implement for local users is crucial in addressing technical and practical challenges related to local people's participation in monitoring (Danielsen et al. 2014, Singh et al. 2014).

Mongolia, with its vast rangelands threatened by changing land use and climate (Liu et al. 2013, Zhao et al. 2015), limited government capacity for formal monitoring, and 4,000‐yr history of nomadic pastoralism rooted in TEK (Fernandez‐Gimenez 2000, Honeychurch 2010), holds potential to serve as a global model for development of integrated rangeland monitoring indicators and participatory community‐based monitoring. A rapidly warming climate (MARCC 2014, Venable et al. 2015), combined with a growing livestock population (Gao et al. 2015), changes in pastoral land use patterns (Fernández‐Giménez et al. 2017), and increased anthropogenic disturbance and fragmentation from mining, road‐building, and urbanization (Dendev et al. 2003, Schweitzerl and Priess 2009), challenge the future sustainability of Mongolia's rangelands. Three nation‐wide rangeland assessments determined that about 65% of Mongolia's rangelands are slightly to severely degraded (Bulgan et al. 2013, NAMEM and MEGDT 2015, Jamsranjav et al. 2018), though much of the affected area could recover within 5 yr with improved grazing management and typical rainfall (NAMEM and MEGDT 2015). The combined effects of increased grazing pressure, changing spatial patterns of grazing, and decreased plant‐available moisture suggest that Mongolian rangelands are at risk and rangeland assessment and monitoring tied to local, regional, and national rangeland management decision‐making is urgently needed. Globally, many of the world's rangelands are in a similarly precarious state (Prince 2016).

**Figure 1:**
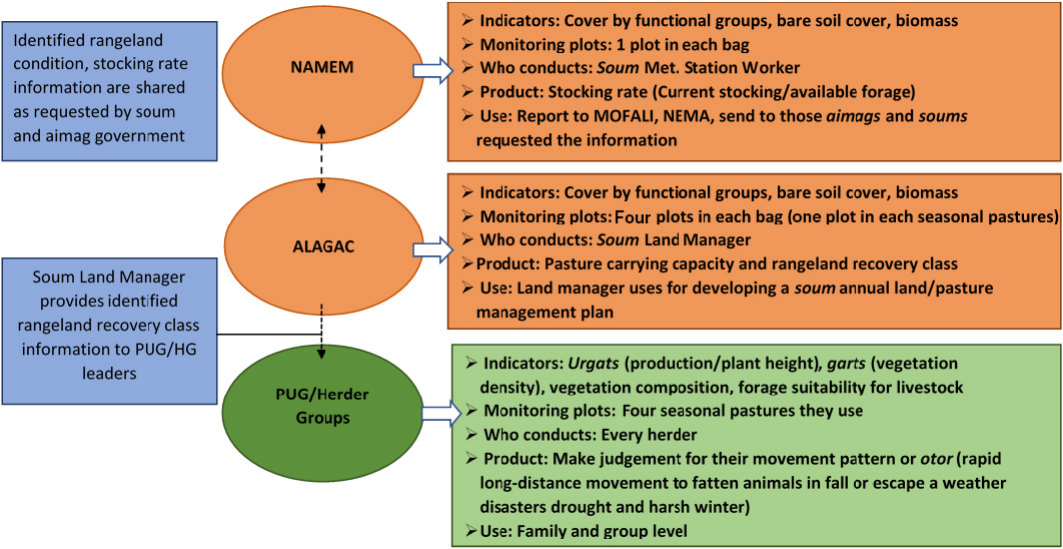
Current one‐way flow of rangeland monitoring information from national government agencies (NAMEM, ALAGAC) to local herder groups in Mongolia. NAMEM, National Agency for Meteorology and Environmental Monitoring; ALAGAC, Administration of Land Affairs; Geodesy and Cartography; MOFALI, Ministry of Food and Agriculture; NEMA, National Emergency Management Agency; PUG, Pasture User Groups.

The Government of Mongolia has implemented a national rangeland assessment and monitoring program, coupled with local rangeland planning, not unlike programs implemented in other countries around the world. However, like elsewhere, there remains a disconnect between Mongolia's formal government‐run rangeland monitoring program, and local government capacity to apply the results to grazing management decisions, which are largely made by herders, as individuals or as groups. Current formal rangeland monitoring programs in Mongolia include the following. First, nationwide rangeland health monitoring is conducted by the National Agency for Meteorology and Environment Monitoring (NAMEM) at the *soum* (district or county) level to assess rangeland condition and identify *soum*‐level stocking rates. This local‐level monitoring occurs with little or no participation by herders. Second, the *soum* land manager or employee of the Agency for Land Administration and Management, Geodesy and Cartography (ALAGAC) conducts monitoring to calculate pasture carrying capacity and rangeland recovery class in each *bag* (sub‐district). Although land managers are meant to provide monitoring results to herders and local government, information flow is slow, and application of monitoring data is inconsistent (Fig. 1). With little or no access to formal monitoring results, herders make decisions about seasonal movements based on TEK and their own assessments of rangeland condition (Fig. 1).

Community‐based rangeland management (CBRM) groups began forming in Mongolia in the late 1990s, in response to declining pasture conditions and rising rural poverty. Members of CBRM groups are more proactive in addressing resource management issues and use more traditional and innovative rangeland management practices, including formal rangeland monitoring, than non‐members (Baival and Fernandez‐Gimenez 2012, Fernandez‐Gimenez et al. 2015, Ulambayar et al. 2017). Yet there are several disconnects between formal government monitoring and rangeland monitoring carried out by CBRM initiatives. First, measurement methods used in formal monitoring typically are not simple enough for herders to apply. Second, formal monitoring does not include indicators that herders use when they assess rangeland conditions, or at least it does not use the same terminology to name them. Third, there is a delay between data collection and reporting findings back to the local government and CBRM groups for use in management decisions. Formal monitoring results are thus not available in a timely and usable form to local herders to support household and community group‐level management decisions.

Currently, CBRM herders are informed about the location where formal monitoring sampling has occurred and sometimes receive monitoring results but are not otherwise involved in actual monitoring activity such as data collection and analysis (U. Budbaatar, *personal communication*). Participatory monitoring by herders using integrated indicators that combine TEK and measures used in formal government monitoring offers a potential means to ameliorate disconnects between formal rangeland monitoring programs and the application of monitoring results by local government and CBRM herders.

A growing number of TEK studies focused on Mongolian pastoralists serve as a starting point. These studies have documented pastoralists’ ecological knowledge and observations of ecological change (Fernandez‐Gimenez 1993, 2000, Bruegger et al. 2014), recorded and compared herders’ observations with ecological field study findings (Kakinuma et al. 2008), and degradation or threshold changes (Kakinuma et al. 2014), and compared herders’ observations of long‐term climate and rangeland changes to remote sensing and meteorological records (Marin 2010, Goulden et al. 2016). As yet, no studies in Mongolia have identified and combined indicators used by herders and scientists to enhance mutual understanding, credibility, and relevance of both scientific and herder observations to inform rangeland assessment, monitoring, and management. Thus, an opportunity to engage herders in collecting, interpreting, and using monitoring data is being missed. Further, there is a risk that if herders do not understand or participate in government monitoring, they will not trust or use the results. Therefore, there is a need for greater understanding of herders’ knowledge and its relationship to formal monitoring indicators and measures. Herders’ participation in formal monitoring could play a vital role in strengthening resource management and supporting adaptation to environmental change.

In light of this potential, our objective is to describe, compare, and integrate herder assessments and indicators of rangeland conditions with field‐based formal monitoring measurements of rangeland conditions at the same sites. We aim to reveal potential complementarities and synergies between these different knowledge systems and identify opportunities to develop integrated monitoring indicators and protocols that are credible, meaningful, and useful to herders, government land managers and researchers.

We ask the following research questions: (1) How do herders assess the ecological condition of rangelands and what indicators do they use? Specifically, what terms and language do they use when describing rangeland health and degradation indicators? (2) According to herders, what factors drive observed differences and changes in rangeland condition? (3) What are the relationships between herders’ assessments of rangeland condition, the indicators they use, and field‐based ecological measurements of the same plots? (4) How well does a multivariate analysis of the factors determining plant community composition align with the herders’ observations of the factors causing vegetation change?

## Methods

### Study sites

**Figure 2:**
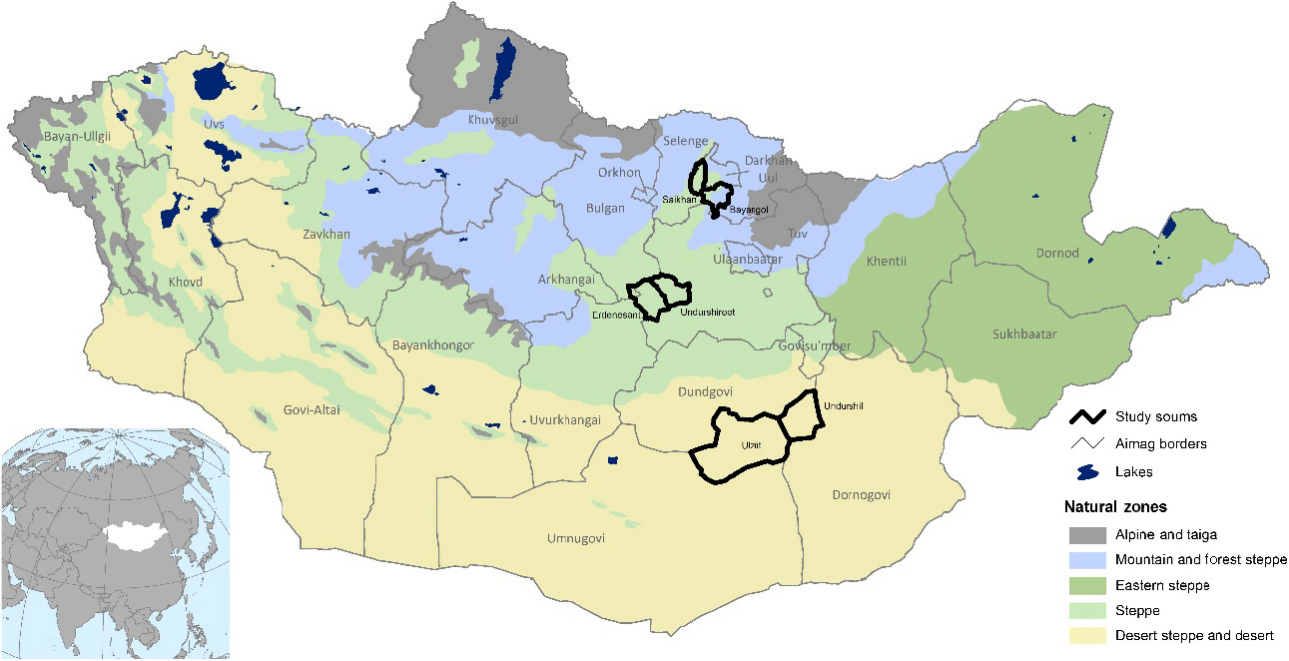
Location of study sites in Mongolia in relation to ecological zones.

We conducted our study in six *soums* (counties) across three *aimags* (provinces) in Mongolia. Study sites were located in three different ecological zones: the mountain and forest steppe (MFS), steppe (ST), and desert steppe (DS) ecological zones (Fig. 2). Mean annual temperature, precipitation, and coefficient of variation (CV) of annual precipitation are −1.1°C, 304 mm, and 25% in the MFS; 0.4°C, 238 mm, and 30% in the ST; and 2.8°C, 123 mm, and 32% in the DS. For each sample plot location, we extracted precipitation and temperature from the Climate Prediction Center (CPC) unified precipitation data set (Chen et al. 2008) and the Global Historical Climate Network Temperature data set (Lawrimore et al. 2011). These ecological zones span a continuum of rangelands from equilibrium (MFS) to non‐equilibrium (DS) dynamics (Fernandez‐Gimenez and Allen‐Diaz 1999, von Wehrden et al. 2012). In arid rangelands with non‐equilibrium dynamics, rainfall amount and variability have greater influence on plant production and species composition than livestock grazing. In moist rangelands with equilibrium dynamics, where precipitation is higher and less variable, biotic factors such as grazing have a stronger influence on plant communities and production (Fernandez‐Gimenez and Allen‐Diaz 1999, Vetter 2005).

Ecological sampling took place in 2011 in the DS and in 2012 in the ST and MFS. Interviews in all zones took place in 2013. In the MFS, total precipitation was similar in the sampling year 2012 (312 mm) and interview year 2013 (306 mm). In the ST, precipitation in the sampling year 2012 (257 mm) was greater than in the interview year 2013 (176 mm). In the DS, sampling year 2011 precipitation (85 mm) was less than in the interview year 2013 (115 mm). We address the implications of these differences in *Discussion*.

### Sampling design, data collection, and data analysis

As part of a larger study (see Ulambayar et al. 2017, Jamsranjav et al. 2018), we surveyed households belonging to four to five herder communities within each study *soum*. For one household in each group, we also sampled vegetation and soils in the winter pasture area used by this household. In this study, we used two different data sources; quantitative vegetation and soil data and in‐depth semi‐structured herder interviews. To examine herders’ assessments of healthy and degraded rangelands and the indicators they use, we conducted herder interviews on the same plots where we sampled vegetation.

#### Vegetation sampling

To capture a spectrum of rangeland conditions, we sampled vegetation along grazing gradients, from heavily grazed pastures near winter livestock shelters to more lightly grazed pastures farther from shelters. We sampled 26 winter pastures in July and August of 2011 and 2012. At each winter shelter, we sampled vegetation in three plots located at 100, 500, and 1,000 m from the winter shelter, as measured from the gate of the shelter corral. Along each gradient, we located plots at the same landform, slope position, and elevation to minimize variation due to edaphic differences. Each 50 × 50 m plot consisted of five systematically spaced 50‐m transects. Transects originated at the 0 point, 12.5, 25, 37.5, and 50 m along the baseline. We measured plant foliar cover by species using the line point intercept (LPI) method (Herrick et al. 2009). All nomenclature follows Grubov (1982). We estimated species richness by walking systematically through the entire 50 × 50 m plot and recording all species observed. We clipped standing crop biomass by functional group in five 0.5 × 0.5 m quadrats in each plot (1 × 1 m in the DS). Biomass was oven dried and weighed in the lab. Total biomass is sum of current year's growth only (litter and standing dead biomass were not included).

#### Herder interviews

We conducted in‐depth semi‐structured interviews with one herder at each winter camp gradient (*n* = 8 in the MFS, *n* = 9 in the ST, and *n* = 9 in the DS) in July and August 2013. Prior to each interview, we obtained free, prior, and informed consent from each individual following our approved IRB protocol (Colorado State University IRB protocol 11‐2514H). The interviewed herder was the traditional “owner” of the camp, and thus was intimately familiar with the ecology, weather, use, and management history of the site. Twenty of the 26 participants were men and six were women. Eleven participants were over 51 yr of age (average age 61 ± 4.8 [mean ± SD]), eight participants between 40 and 50 yr old (average age 44.9 ± 3.2), and seven participants were younger than 40 yr old (average age 35.7 ± 4.7). Interviewed herders varied in the number of years they had used the sampled winter shelter and surrounding pasture. The average duration of use was 17 yr, with a range from 3 to 50 yr. Interviews were guided by a questionnaire consisting of three sections, including household information, definitions of rangeland health and degradation and causes of degradation, and perceptions of vegetation conditions at the three plots at their winter shelter (See Appendix S1). In the household information section, we asked about the respondent's age, gender, length of use of this winter shelter, number of families using the shelter, livestock numbers, and out‐of‐season grazing. We recorded livestock number by each species of all families using the winter camp, including the camp owner and other families camping with them and grazing their livestock in the same, shared pasture. We converted different species into sheep forage units (SFU), the livestock equivalency unit used in Mongolia, where 1 camel = 5 SFU, 1 horse = 7 SFU, 1 cow/yak = 6 SFU, 1 sheep = 1 SFU, and 1 goat = 0.9 SFU (NSO 2013). The second section of the questionnaire asked herders what indicators they use to identify healthy and degraded rangelands and perceived causes of rangeland health and degradation. For the third section, we brought herders to each of our three study plots near their winter camp where we sampled vegetation and soils and asked them to evaluate the condition of each plot. We asked herders to rate the overall condition of each plot using a meter stick with a scale from very healthy pasture (40) to very unhealthy pasture (0). Then we asked the herder what specific indicators they used to judge the condition of the plot and to rank the indicators from the most important to least important indicator. The interviewer (C. Jamsranjav) is a native Mongolian speaker and all interviews were conducted in Mongolian. Interviews were audio‐recorded.

#### Analysis of herder interview data

Some of the interview data were closed‐ended and quantitative and some were open‐ended and qualitative. We entered close‐ended quantitative interview data into an Excel (for Windows, MS Office) spreadsheet for further analysis using SAS (version 9.4 for Windows, SAS Institute, Cary, North Carolina, USA) and PC‐ORD (version 6 for Windows, McCune and Mefford 2011) software. We transcribed audio recordings of the qualitative open‐ended interview responses into a Word (for Windows, MS Office) file in Mongolian. Transcribed interviews were imported into NVIVO (2015) for coding. First, we developed an a priori list of codes based on the interview questions. The Mongolian text was coded using codes (categories) named in English. We organized coded passages into tables to compare and synthesize responses within and between ecological zones, translating coded text from Mongolian into English for this analysis. The authors discussed extensively the meaning and appropriate translation of specific monitoring indicators from Mongolian to English. In reporting our results, we retain key Mongolian terms together with our English translations, to ensure transparency and fidelity, and to allow other Mongolian readers to challenge our translation. Second, in synthesizing the results, we aimed to characterize how herders look at and talk about rangeland conditions and degradation, the indicators they use, and what these indicators signify to herders as they assess rangeland conditions at different grazing intensities. We assessed the importance of different indicators and causes of rangeland health and degradation by calculating the frequency with which different indicators and causes were mentioned across interviews.

#### Quantitative analysis of field data and herder assessment scores

We used linear regression to determine relationships between vegetation variables measured at the plots and herders’ assessment scores on those plots. Vegetation variables included in the linear regression were total foliar cover, palatable plant cover, species richness, basal cover, litter cover, total biomass, grass biomass, forb biomass, shrub biomass, sedge biomass, and litter biomass. We used these measured indicators because they most closely approximated the qualitative indicators that herders discussed in their interviews (see *Results*). The statistical package SAS 9.3 for Windows (SAS Institute) was used in this analysis.

We used cluster analysis to identify distinct plant communities within each ecological zone, and non‐metric multidimensional scaling (NMS) ordination to relate plant species composition to measured environmental variables and to herders’ quantitative assessment scores and their qualitative indicators and perceived drivers of rangeland conditions. This quantitative approach builds on earlier work by Reed et al. (2008) and Roba and Oba (2009). This approach provides an opportunity to deepen our understanding of how herders understand and interpret plant communities in relation to rangeland conditions and environmental factors. Further, it helps to identify potential complementarities and synergies of using herders’ ratings of plot conditions and the indicators they discussed in interviews for integrated indicators and participatory monitoring. We used agglomerative hierarchical cluster analysis to identify potential plant communities. We used Sorensen similarity, also known as the Bray‐Curtis (Bray‐Curtis) distance measure, to calculate distances among sample plots in species space and flexible beta linkage method (β = 0.25), which is conceptually compatible with Sorensen (McCune and Grace 2002). All species (rare and common) were included in the classification analysis. We used indicator species analysis to describe the value of different species for indicating environmental conditions. This method combines information on species that are concentrated and abundant in a particular group and faithfulness of occurrence of a species in a particular group of plots (Dufrene and Legendre 1997). The indicator value generated was between 1 and 10 based on the faithfulness and exclusiveness of species to groups of plots. We selected the number of groups with the most significant indicator species (lowest average *P* for species based on a randomization test with 4,999 randomizations; McCune and Grace 2002). We entered the grouping variables from the classification into our ecological data file. We used one‐way ANOVA followed by Tukey‐adjusted multiple comparison tests to assess differences among community groups within each ecological zone in total biomass and biomass by functional group. We entered the grouping variables from the classification analysis into our environmental variable matrix for the ordination. Then we used NMS to describe the relationship between plant species composition, environmental variables, and herders’ evaluation of plots. The grouping variables are used to show community group membership in the NMS diagrams. We ran NMS from random starts with medium thoroughness settings for autopilot mode in PC‐ORD (Version 6) using same Sorensen distance method. Because the effects of ecological zone were large, we ran the analysis separately for each ecological zone.

## Results

### Herders’ definitions of healthy vs. degraded rangeland

#### Indicators and causes of healthy rangeland

Herders use four main indicators to define rangeland health: forage suitability for livestock (*maldaa tohirson ideeshlegtei*) or good forage/palatable perennial plants for livestock (*mald targa avakhuualkh/mal idekh durtai, urgamaltai*), plant species diversity (*olon turliin urgamaltai*) and composition, plant density (*garts*), and plant growth or production (*urgats*; Table 1). As described by herders, forage suitability and presence of good forage/palatable plants are similar indicators but differ in the specificity of observations. Herders explained that forage suitability is assessed holistically at the pasture scale, whereas good forage/palatable plants for livestock is assessed by observing presence, absence or abundance of specific plant species in a pasture or patch. Herders define *garts* as the number of individual plants that emerge or grow when spring comes. Thus, *garts* refers to the density of plants not shoots or tillers. Herders explained that when *garts* is poor, few plants will grow even if there are roots in the soil. *Urgats* means plant growth which is assessed by vegetation height and cover, and is associated with overall plant production. In addition to the indicators mentioned above, herders in all zones focus on the abundance of tender perennial grass species that they refer to as thin grass or *nariin uvs* such as *Festuca* spp. and *Poa* spp. in the MFS, and *Stipa* spp. in the ST and DS zones. Finally, herders in the ST and DS value palatable subshrubs and shrubs such as *Artemisia frigida*,* Kochia prostrata*, and *A. xerophytica*.

**Table 1:** Indicators used by Mongolian herders in different ecological zones to evaluate healthy and degraded rangelands and observed causal factors

Category	Mountain and forest steppe (MFS) (*n* = 8)	Steppe (ST) (*n* = 9)	Desert steppe (DS) (*n* = 9)
Indicators of healthy rangelands	plant species diversity and composition (4), high plant density (*garts*) (3), good forage plants for livestock (*mal idekh durtai*) (3), presence of litter (2)	good plant growth (*urgats*) (6), high plant density (*garts*) (4), good forage plants for livestock (*mal idekh durtai*) (4), presence of litter (2)	forage suitability for livestock (*maldaa tohirson ideeshlegtei*) (5), plant species diversity (*olon turliin urgamaltai*) and composition (4), high plant density (*garts*) (3), good plant growth (*urgats*) (2)
Causes of healthy rangelands	livestock numbers within pasture carrying capacity (5), good regular rainfall (4), pastures rested and rotated so they recover and regrow (3)	good regular rainfall events (8), frequent livestock movements, rest and rotation (4), livestock numbers within pasture carrying capacity (3)	Summer and late fall precipitation (8)
Indicators of degraded rangelands	low plant density (*garts*) (4), low plant vigor (*siireg urgamal*) (4), weedy unpalatable plants dominate (4), abundant bare ground (3), few plant species (2)	abundant bare ground, hard soil (8), weedy unpalatable plants dominate (5), few plant species (3), low plant vigor (*siireg urgamal*) (2)	extensive exposed or dead plant roots (6), few good palatable plant species (6), increased sand movement (4), weedy unpalatable plants dominate (3), low plant vigor (*siireg urgamal*) (3)
Causes of rangeland degradation	increased livestock numbers (8), less summer rain (3), no resting and rotating rangelands (2)	out of season grazing (i.e., grazing winter pastures in summer) (5), exceeding pasture carrying capacity (4), trampling by livestock hooves (3)	changes in timing and spatial distribution of rainfall (8), reduced total amount of rainfall (7), increasing dust storms (5)

*Notes*

Indicators are listed in order from the most frequently to least frequently mentioned indicator in each zone. Numbers indicate how many herders mentioned that indicator.

The most frequently mentioned indicators by herders in the mountain and forest steppe (MFS) were the number of different plant species and the types of plants (diversity and community composition), followed by plant density or *garts*, the presence of good forage/palatable plants for livestock, and presence of litter. ST herders most frequently mentioned vegetation growth or *urgats*, plant density or *garts*, good forage/palatable perennial plants for livestock, and presence of litter as indicators of rangeland health. Most DS herders mentioned forage suitability for livestock most frequently, followed by plant diversity and composition, plant density or *garts*, and vegetation growth or *urgats* last. DS herders emphasized that forage suitability for livestock is important and different plant types provide different flavors and nutrients. Herders elaborated that livestock on pastures with a diversity of plants and high quality forage plants graze longer in one patch, which contributes to livestock weight gain. As a DS herder explained, “If there are many different types of plants, then the pasture can feed more livestock, especially grasses are very good forage during winter, also shrub, and subshrubs such as *Artemisia xerophytica* is good forage plant for livestock and adds flavor to livestock food. If there is only one type of plant, then livestock get bored and they don't graze long enough at the pasture, move frequently from one patch to another.” Additionally, pastures with high plant diversity provide livestock forage during different seasons and environmental conditions. For example, although *Allium* species (e.g., *A. polyrrhizium, A. mongolicum*) are good spring, summer, and fall forage, these plants have little forage value during droughts or winter unless they are harvested and dried to make supplemental feed. In contrast, shrubs such as *Salsola passerina*,* Saussurea amara*, and *A. xerophytica* and desert grasses of the *Stipa* genus are drought resilient and retain nutritional value during winter, according to herders.

MFS herders identified three main factors that contribute to rangeland health, in order of frequency of mention: keeping livestock numbers within pasture carrying capacity, resting and rotating pastures so they regrow and recover, and good, regular rainfalls. Herders explained that “good” rain is rainfall with abundant water but not a high‐intensity rainfall event. In the words of one herder, “If we have regular and soft rain and warm sunny days in summer, then livestock hooves won't trample the soil to powder.”

ST herders indicated that the main factors contributing to healthy rangelands (in order of frequency of mention) are good rainfall, frequent herd movement (pasture rotation), and not exceeding pasture carrying capacity. As a ST herder explained, “Don't graze many livestock in the same place. Herders should live/stay in a more scattered way and move frequently to rotate grazing. Herder families also should not graze or send livestock in the same direction every day.” A few ST herders emphasized that landform and slope position influence rangeland health. Usually areas that have snow accumulation or sheltered places have good vegetation in spring and summer and soils are less prone to damage by livestock hooves.

DS herders explained that summer rainfall and late fall precipitation are the main factors influencing rangeland health in their zone. In spring, plants begin to grow in places that had late fall precipitation. These early emerging green plants are important food for livestock to gain strength after the long winter of eating mostly standing dead biomass. Early summer precipitation contributes to emergence of diverse plant types, which contribute to livestock weight gain.

#### Indicators and causes of degraded rangeland

Herders in the mesic, productive MFS identified degraded rangeland based on low density of plants (*garts*), low vegetation vigor (*siireg or utgun*), and low plant diversity with communities dominated by weedy, less palatable plants (*umkhii sharilj*,* Artemisia pectinata*;* shivee*,* Stipa cappillata*;* khalgai*,* Urtica* species; *tuiplantsar*,* Phlomis* species). Herders discuss plant vigor in terms of *utgun* and *siireg* plants, when observing the number of leaves, tillers, or branches on an individual plant. Few leaves or branches on a single plant indicate a *siireg* or low‐vigor plant and more leaves and branches indicate an *utgun* or vigorous plant. ST herders use the area of bare ground and soil hardness as degradation indicators, in addition to observing dominance of weedy and unpalatable plants on pastures. DS herders also use soil‐related indicators, observing signs of erosion, such as the extent to which plant roots are exposed or have died, and sand movements. DS herders also looked at the proportion of palatable forage plants and weedy plants in the pasture to assess degradation.

As for causes of rangeland degradation, MFS herders described increased livestock numbers or exceeded carrying capacity, failure to rest and rotate rangelands, and poor summer rain as the main causes of poor grassland conditions. Herders in this region emphasized that government policy is essential for rangeland protection and management. One elder herder emphasized, “In Mongolia, we are doing extensive herding. Therefore, rangeland protection and management are very critical. The government is paying attention to the number of bales of hay to be used in winter, not about regulating pasture use, resting rangeland, and rotating livestock, which is very essential for overcoming harsh winters. In the old days, we used to have four to five households as a khot ail (herding camp), livestock grazing orbit was about 4–5 km, which means we saved that size of pasture for winter. Our livestock overcame winter just by grazing on the pasture. But nowadays, anybody can come and graze or collect hay as long as they stay away at least 100 m from my winter shelter. I cannot say anything if someone with 200 horses comes and grazes on my winter pasture. Therefore, I blame the government, they are not working to regulate pasture use and help herders to overcome harsh winters.”

ST herders identified the main causes of rangeland degradation as out‐of‐season grazing, exceeding carrying capacity, continuous grazing in one place, and trampling of rangelands by livestock hooves (literally, turning the soil to powder, *talhlakh*). Other contributing causes mentioned by a few herders were very hot sun and increasing rodent populations (i.e., Brandt's vole). Herders refer to burned vegetation (*shatsan nogoo*) when vegetation dries and dies under extremely hot sun. Herders report that too many rodents collect grass for building their underground nest or food hoard, resulting in degradation.

DS herders saw the main causes of rangeland degradation as changes in the temporal and spatial distribution and the total amount of precipitation and spring dust storms. For example, herders observed that rain events are becoming patchier in this region. “Lately, we have been having less rain and the rain is becoming patchy, we can have rain here and there. For example, we have rain here but there is no rain even within 2 km away from our place. So, then people move to the place that had rain, which creates more livestock congregation in a small area and therefore contributes to rangeland degradation eventually.” DS herders also agreed that early winter and summer precipitation are declining and occur later in the season compared to the past. Herders see early summer precipitation as essential for protecting soil and vegetation roots from dust storms. Gobi herders observed that vegetation is increasingly sparse due to delays in rains that start the growing season. Consequently, spring dust storms transport more soil and expose and damage plant roots. Herders observed that in areas hit by spring dust storms, weedy species dominate after late summer rains. As one herder recounted, “If we don't have rain, then there will be no vegetation. If there is no vegetation, then wind and dust storms destroy plant roots, which causes severe rangeland degradation.”

### Relationships between herders’ assessments and quantitative plot‐based measurements

#### Herders’ assessments of plots

When we brought each herder to each of three study plots near their winter camp, we asked them to give their ratings on overall condition of each plot using a stick marked with a scale from very healthy pasture (score = 40) to very unhealthy pasture (score = 0). After the herder assigned an overall quantitative condition rating using the stick, we asked them to describe the indicators they used to arrive at their overall assessment of plot condition. Across all ecological zones, herders gave the lowest average condition scores to the heavily used 100‐m plots (MFS = 19, range 10–30; ST = 18.4, range 6–30; DS = 18.4, range 5–30). In the ST and DS, herders gave the highest mean condition scores to lightly used 1,000 m plots and in the MFS the highest scores were for the 500‐m plots with intermediate use levels (mean score for the 500‐m plots, MFS = 24.8, range 10–40; ST = 23.1, range 10–35; DS = 25, range 7–40; mean score for the 1,000‐m plots, MFS = 23.1, range 10–40, ST = 26, range 6–40; DS = 25.6, range 10–40). Interestingly, MFS herders believed that all plots with low scores can return to good condition (score = 40). One ST herder disagreed that a low scored ST plot could regain a high condition score. Two DS herders answered that their plots at 100 and at 500 m (one herder) could never return to the highest condition score.

Herders explained that the poor condition of low‐scoring plots were caused by the proximity of plots to livestock impact points such as shelters or wells, intensive livestock grazing and trampling, low soil moisture conditions, and high intensity and direction of wind that affects the plot and soil physical structure. Herders explained that most 500‐ and 1,000‐m plots are in better condition (compared to 100‐m plots) and they assigned higher scores to these plots. According to herders, the 500‐ and 1,000‐m plots are in better condition because they are located farther from livestock impact points and receive less impact from trampling and grazing. Herders also explained that at these more distant plots, the soil is softer (less compacted) and therefore these plots absorb rain and snow water better. When herders assigned low scores to 500‐ and 1,000‐m plots, they attributed poor conditions to poor soil moisture due to late or poor rain/snow events.

#### Linear regression

Based on herders’ identified indicators in each zone, we selected the corresponding vegetation and soil variables collected during our field sampling that most closely approximated herder indicators and regressed these against herders’ condition ratings to assess how well herders’ ratings corresponded with our field measurements of the same indicators. We selected five ecological variables that were closest to the indicators that herders described in the interviews. We selected species richness as comparable with herders’ identified indicator of plant diversity and composition, total foliar cover as comparable with herders’ identified indicator of plant density (*garts*), cover of palatable plants as comparable with herders’ identified indicator of presence of good forage plants, and total biomass as comparable with herders’ identified indicator of plant growth (*urgats*), and litter cover as comparable with herders’ identified indicator of presence of litter on the pasture.

**Figure 3:**
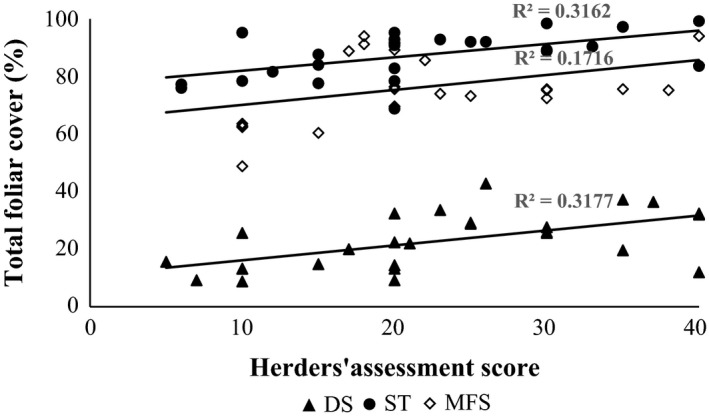
Linear regression of total foliar cover of plots against herders’ quantitative assessment scores (0–40) of plot condition in three ecological zones (DS, desert steppe; ST, steppe; MFS, mountain and forest steppe).

Herders’ assessment scores in all zones were significantly positively correlated with measured total foliar cover (Fig. 3). However, the correlation was weak in the MFS due to a cluster of points with low herder scores and high cover values. These six points were from two gradients where one of the herders explained his low scores based on the abundance of *buduun uvs* or “thick grass” of lower nutritional quality. *C. duriuscula* and/or *C. korshinskii*, low palatability sedge species known to increase with grazing pressure, were common on all the outlier plots and may explain the low herder scores. In addition, MFS zone herders’ scores were positively and significantly correlated with measured grass biomass (*P* = 0.01; *r* ^2^ = 0.26). ST zone herders’ scores were significantly and positively associated with cover of palatable plants (*P* = 0.0006, *r* ^2^ = 0.38) and total biomass (*P* = 0.04, *r* ^2^ = 0.15). DS herders’ scores were positively and significantly correlated with cover of palatable plants (*P* = 0.0008, *r* ^2^ = 0.37), species richness (*P* =0.006, *r* ^2^ = 0.26), total biomass (*P* = 0.01, *r* ^2^ = 0.22), grass biomass (*P* = 0.008, *r* ^2^ = 0.25), and litter cover (*P* = 0.02, *r* ^2^ = 0.15).

#### Classification and ordination

We used cluster analysis and nonmetric multidimensional scaling (NMS) to describe the relationship between plant species composition, measured environmental variables (mean growing‐season precipitation, mean yearly rainfall precipitation from 1979 to 2012, slope, aspect, elevation and soil texture), number of livestock (in sheep forage units), distance of each plot from winter shelter, and herders’ assessment scores for each plot. We conducted this analysis for two reasons. First, we wanted to compare herder assessment scores and qualitative indicators related to plant community composition to plant community composition derived from our measurements of species cover, to deepen our understanding of how herders’ perceptions of plant communities relate to how researchers classify and interpret these communities. Second, we wanted to relate the causes of rangeland health and degradation described by herders, to plant community responses to grazing and other environmental variables determined by ordination. Here, we report the results of classification and ordination by each ecological zone in turn, starting with the MFS.

Seventy‐two species were included in the MFS classification and three community groups were identified as follows (see Appendix S2: Table S1 for complete descriptions of each group): community group 1, *Cleistogenes squarrosa*/*Artemisia frigida/Carex duriuscula* (*n* = 12); community group 2, *Poa attenuata*/*Carex korshinskyi* (*n* = 4); and community group 3, *Agropyron cristatum*/*Allium senescens*/*Caragana microphylla* (*n* = 8). Dominant species of community group 1 are known to be grazing tolerant (*C. squarrosa, S. krylovii*) and disturbance indicator species that increase with moderate to heavy grazing (*C. duriuscula*). Dominant grasses of community group 2 (*P. attenuata*,* Festuca lenensis*) are highly palatable and decrease with heavy grazing. Codominants in this community group include a wide variety of forbs typical of cooler MFS environments (*Dianthus versicolor*,* Vicia cracca*,* Thalictrum simplex*,* Bupleurum bicaule*,* Saussurea salicifolia*, and *Galium verum*). Dominant species of community group 3 (*A. cristatum*/*A. senesces*/*C. microphylla*;* n* = 8) are grazing tolerant and are classified as increasers, species that increase with moderate grazing pressure and decrease with very heavy grazing. In the MFS, grass biomass was significantly greater for plots in community group 3 than for plots classified in community group 1 and 2 (*P* = 0.002). Plots of community group 3 had higher cover of palatable plants than plots of community group 1 (*P* = 0.03). Community group 2 plots had higher species richness than community groups 1 and 3 (*P* = 0.0004; see Appendix S5: Table S1 for summary information of each group).

**Figure 4:**
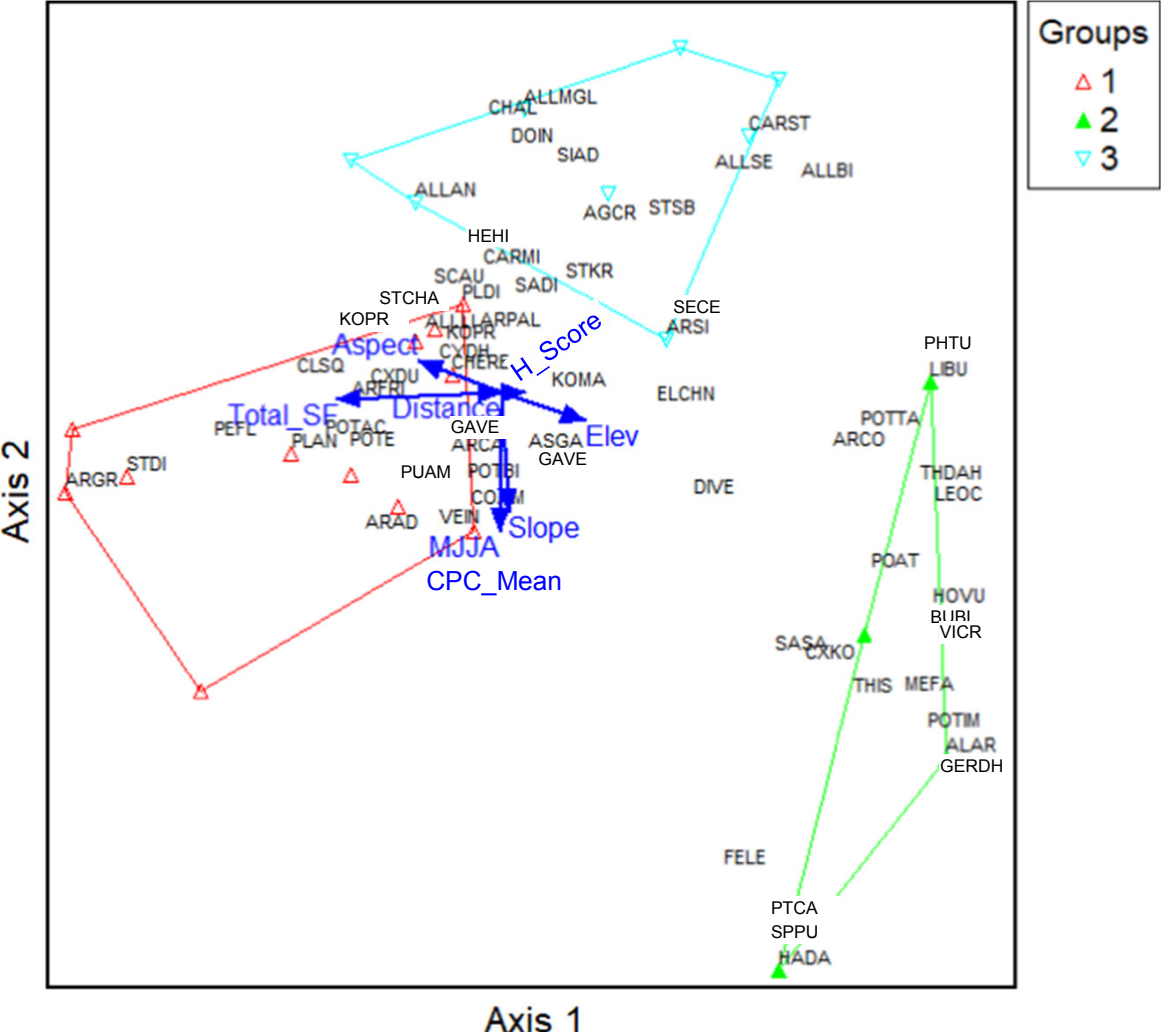
Nonmetric multidimensional scaling (NMS) for the MFS zone. The three groups are potential plant communities identified from the agglomerative cluster analysis (see Classification and ordination). Arrows indicate abiotic and biotic variables highly correlated with NMS axes and explain most of the variation in species composition. Total livestock number by sheep forage unit (SFU) is correlated with Axis 1 (*r* = −0.60). Axis 2 is highly correlated with growing‐season (MJJA, May–August) precipitation of the year we collected the vegetation data (*r* = 0.55), yearly mean rainfall precipitation from 1979 to 2012 (CPC_mean; *r* = 0.55) and slope (*r* = 0.50). See Appendix S1 for key to species name abbreviations.

In the MFS, plant species composition was most strongly correlated with total number of livestock in sheep forage units (SFU; *r* = −0.60), elevation (*r* = 0.43), and aspect (*r* = 0.43; Fig. 4). Herders’ assessment scores correlated with axis 1 (*r* = 0.23) and the direction of the vector was opposite to the total livestock number, such that high herder assessment scores corresponded with low livestock numbers. Growing‐season precipitation and slope were highly correlated with axis 2 (*r* = 0.55 and *r* = 0.50). In this zone, the most important drivers of plant community composition were livestock use and precipitation. Plant communities are arrayed from left to right along axis 1 from community 1 with the most disturbance‐associated species, to community 3 with grazing tolerant species, and at the far‐right community 2, with the most grazing‐sensitive species and a diversity of forbs that favor cool and moist sites.

Fifty‐two species were included in the ST and five community groups were identified (see Appendix S3: Table S1 for complete descriptions of each group): community group 1 (*Cleistogenes squarrosa*/*Carex duriuscula*/*Allium polyrrhizum*/*Artemisia frigida*;* n* = 10), community group 2 (*Stipa krylovii*;* n* = 9), community group 3 (*Agropyron cristatum*/*Artemisia adamsii*/*Chenopodium album*‐dominated plot; *n* = 3), community group 4 (*Stipa gobica*/*Kochia prostrata*‐dominated plots; *n* = 3), and community group 5 (*Elymus chinensis* [known as *Leymus chinensis* in China]; *n* = 2). Community groups 2 and 4 include grasses (*S. gobica*) and forbs (*A. polyrrhizum*) typical of the DS, and likely represents the ecotone of the ST and DS regions. Community groups 1, 3, and 5 include grazing‐tolerant grasses (*C. squarrosa, A. cristatum, E. chinensis*), sedges (C*. duriuscula*), and forbs (*C. album, A. adamsii*) that increase with grazing pressure. In this zone, there were no significant differences in total biomass and biomass by functional groups among community groups. Community group 5 had more litter biomass than community groups 1 and 4 (*P* = 0.02; see Appendix S6: Table S1 for summary information of each group).

**Figure 5:**
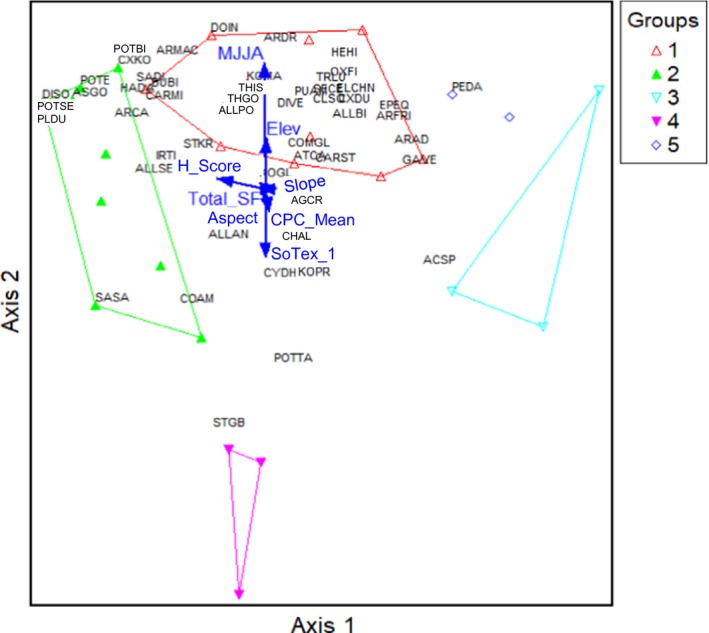
Nonmetric multidimensional scaling (NMS) for the ST zone. Group numbers in the legend refers the potential plant communities identified from agglomerative cluster analysis (see Classification and ordination). Arrows indicate abiotic and biotic variables highly correlated with NMS axes and explain most of the variation in species composition. Herders’ score for the plot condition is highly correlated with Axis 1 (*r* = −0.40) and growing‐season (MJJA, May–August) precipitation of the year we collected the vegetation data, and elevation are highly correlated with Axis 2 (*r* = 0.67 and *r* = 0.42). See Appendix S2 for full species names.

In the ST, growing‐season rainfall and elevation were the strongest drivers of community composition, both positively correlated with axis 2 (*r* = 0.67 and *r* = 0.42, respectively). Axis 1 was correlated with slope (*r* = 0.19) and total livestock numbers (SFU; *r* = −0.13; Fig. 5). Herders’ assessment scores correlated with the main axis (*r* = −0.40) and herders tended to give higher scores to plots dominated by *S. krylovii*. Interestingly, herders’ scores and livestock use were positively correlated, indicating that herders gave higher scores where there was more livestock use.

In the DS, 37 species were included in the classification and four community groups were identified (see Appendix S4: Table S1 for complete descriptions of each group): community group 1 (*Allium mongolicum*/*Salsola collina*;* n* = 15), community group 2 (*Allium polyrrhizum*/*Caragana stenophylla*;* n* = 4), community group 3 (*Stipa gobica*/*Allium mongolicum*;* n* = 5), community group 4 (*Eragrostis minor*/*Dontostemon integrifolius*;* n* = 3). In the DS, grass biomass was significantly greater in community group 3 than in community group 1 (*P* = 0.003). Cover of palatable plants was greater in group 3 than in group 1 (*P* = 0.02) and cover of unpalatable plants was greater in community group 4 were greater than in the other three community groups (*P* < 0.001; see Appendix S7: Table S1 for summary information of each group).

**Figure 6:**
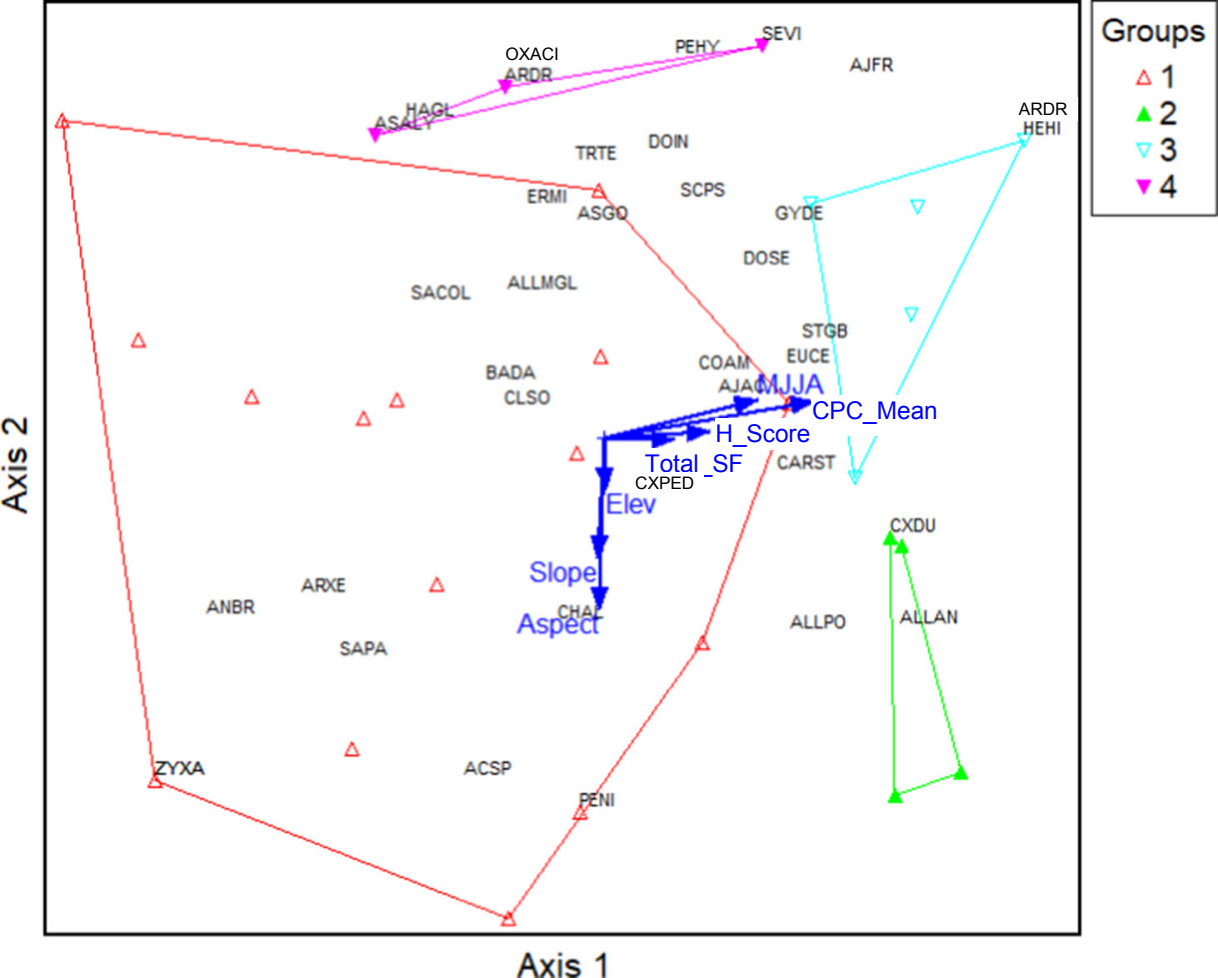
Nonmetric multidimensional scaling (NMS) for the DS zone. Group numbers in the legend refers the potential plant communities identified from agglomerative cluster analysis (see Classification and ordination). Arrows indicate abiotic and biotic variables highly correlated with NMS axes and explain most of the variation in species composition. Mean yearly rainfall from 1979 to 2012 (CPC_mean), growing‐season (MJJA, May–August) precipitation of the year when vegetation data was collected and herders’ score for the rangeland condition are highly correlated with Axis 1 (*r* = 0.66, *r* = 0.56 and *r* = 0.47 accordingly); aspect and slope are highly correlated with Axis 2 (*r* = 0.60 and *r* = 0.48 accordingly). See Appendix S3 for full species names.

In the DS, plant species composition was driven by abiotic factors, primarily precipitation. Axis 1 was correlated with long‐term yearly mean rainfall (*r* = 0.66) and growing‐season rainfall (*r* = 0.56). Herders’ assessment scores were also correlated with axis 1 (*r* = 0.47; Fig. 6). Aspect (*r* = 0.61) and slope (*r* = 0.49) correlated with axis 2. As in the ST, herders’ assessment scores and total livestock number were positively correlated with axis 1 and with each other, such that high assessment scores were associated with high livestock numbers.

## Discussion

### Herder indicators

In‐depth on‐site interviews with herders demonstrate that herder knowledge can be a rich source of information for identifying indicators and potential causes of rangeland health and degradation. Most MFS herders paid attention to plant species diversity and composition when defining rangeland health, which is similar to findings for sheep ranchers in the western United States (Knapp and Fernandez‐Gimenez 2009), but contrasts with herders in some other mesic mountain environments who pay less attention to individual plant species (Fernandez‐Gimenez and Fillat 2012, Molnar 2017). ST herders were more similar to the European herders studied by Fernandez‐Gimenez and Fillat (2012) and Molnar (2017), and most focused on vegetation growth or production (*urgats*) and plant density (*garts*). DS herders identified the presence of good forage plants for livestock most often as an important indicator of rangeland health. This could be because herders in the sparsely vegetated DS zone focus on forage quality over vegetation quantity as an indicator of healthy pasture. This finding is consistent with what African pastoralists observe (Roba and Oba 2009, Jandreau and Berkes 2016).

Declines in suitable forage species and increases in weedy unpalatable plants are the most common herder indicators of degradation in all zones, reinforcing previous studies in Mongolia (Fernandez‐Gimenez 2000, Bruegger et al. 2014) and elsewhere (Mapinduzi et al. 2003, Knapp and Fernandez‐Gimenez 2008, Roba and Oba 2009, Hopping et al. 2016, Jandreau and Berkes 2016). Exposed bare ground was another common herder indicator, which is consistent with other studies in the ST and DS of Mongolia (Fernandez‐Gimenez 2000, Bruegger et al. 2014) and in Africa (Stringer and Reed 2007, Reed et al. 2008). In summary, our findings show promise for developing integrated indicators that incorporate important herder‐observed indicators, such as plant density (*garts*), growth (*urgats*), plant community composition (presence of nutritious or weedy plants, diversity of plant species, etc.), and soil indicators (bare ground, exposed roots), and typical formal rangeland assessment measurements such as total foliar cover, biomass, cover of palatable plants, species composition and richness, and bare ground.

### Relationship between herders’ assessments of rangeland condition and field‐based ecological measurements of the same plots

Most of the approaches to integrating TEK and scientific knowledge focus on how TEK can complement weaknesses of scientific monitoring or vice versa. TEK may fill information gaps such as understanding landscape heterogeneity, improving temporal and spatial resolution of observations, adding detailed information to improve understanding about processes related to degradation, and interpreting management implications specific to certain locations (Stringer and Reed 2007, Roba and Oba 2009, Reed et al. 2013, Klein et al. 2014). Most importantly, integrating TEK and scientific knowledges may help empower local resource users and improve management effectiveness. By involving resource users in decision‐making that affects their livelihoods (Huntington 2000), participatory monitoring that integrates traditional and scientific knowledges can contribute toward sustainability (Tengӧ et al. 2017).

Despite the potential benefits of combining TEK and scientific measurements, approaches for developing integrated indicators, especially quantitative indicators, are lacking (Knapp and Fernandez‐Gimenez 2009, Herrick et al. 2010). In this study, the similarities in the indicators, and the positive correlations between herders’ quantitative assessment scores and field measurements suggest that herders and researchers are observing similar attributes, often interpreting them in similar ways, and arriving at similar overall results or conclusions, but using different terms to name these indicators. By different terms, we refer to differences in the indicator names used by Mongolian herders and those used by Mongolian researchers and monitoring officials. These commonalities suggest that there is a strong foundation for developing mutually understood indicators that are credible and relevant to both herders and researchers or government agencies. At same time, in our study, herders from all zones used terms such as *urgats* (plant growth or production), *garts* (plant density), *shingen urgamal* (low‐vigor plant), and *utgun urgamal* (vigorous plant), which have been mentioned only occasionally in previous TEK studies conducted in Mongolia. These terms were very common among herders in our Mongolian study sites for daily information exchange about rangeland health and degradation. Further steps to extend this research could be to (1) calibrate herders’ qualitative assessments and quantitative scores with the formal government monitoring plots in each *soum* and (2) test the repeatability of herders’ quantitative assessment scores over time to determine if they can be used to detect changes over time as well as differences across space. Because our inferences are limited to the winter pastures where we conducted sampling, a second step is to evaluate our methods in other seasonal pasture areas and determine whether herders’ observations and assessments apply more broadly to all seasonal pastures.

### Perceived causes of rangeland health and degradation, relationship between plant community composition, environmental drivers and herder assessment scores

Herders’ identified causes of rangeland health and degradation were consistent with predictions of equilibrium and non‐equilibrium rangeland vegetation dynamics theory (Ellis and Swift 1988, Fernandez‐Gimenez and Allen‐Diaz 1999), and imply that herders hold tacit mental models of rangeland change, including the reversibility or irreversibility of change. In mesic rangelands where rainfall is higher and inter‐annual variability in rainfall is lower, equilibrium dynamics prevail, and grazing has a stronger influence on vegetation than precipitation. In contrast, non‐equilibrium theory predicts that in arid and highly variable rangelands, abiotic factors such as rainfall variability drive plant production and community composition, and livestock grazing plays a relatively small role. In line with this theory, most MFS herders identified keeping livestock numbers within local rangeland carrying capacity as an important factor contributing to rangeland health and agreed that increasing livestock numbers and failure to rest and rotate rangelands are the main causes of rangeland degradation. Further, all MFS herders believed that sites with low scores could return to good condition with reduced stocking.

In the MFS, plant community groups displayed in NMS ordination space exhibit the typical pattern of vegetation change in response to increasing grazing pressure in equilibrium systems. The most important drivers of MFS plant community composition were livestock use and precipitation. The ordination illustrates that herders’ assessment scores are strongly correlated with the dominant axis of community composition suggesting that herders are indeed observing the same differences in community composition that we measured in the field. Our finding aligns with other TEK studies conducted in this mesic and more productive zone of Mongolia, where herders also identified heavy grazing is the primary factor influencing rangeland degradation (Kakinuma et al. 2008, Bruegger et al. 2014). Herder observations in our study and others are consistent with scientific observations that livestock grazing has a strong effect on rangeland condition in this zone (Fernandez‐Gimenez and Allen‐Diaz 2001, Van Staalduinen et al. 2007, Jamiyansharav et al. 2018).

Similarly, conforming with non‐equilibrium rangeland dynamics theory, DS herders attributed rangeland health primarily to rainfall and saw the temporal (decreasing summer and late fall precipitation) and spatial distribution of rainfall and reduced total amount of rainfall as major drivers of degradation. This observation was supported by the ordination analysis, where the most important drivers of DS plant community composition were long‐term mean yearly rainfall and growing‐season rainfall. Several DS herders believed that sites with low condition scores could never recover fully, indicating potentially irreversible changes. Interestingly, DS herders’ assessment scores were positively correlated with livestock use, not negatively correlated as in the MFS. Herders’ assessment scores and total livestock number were positively correlated with each other and with axis 1. It is likely that herders give high assessment scores to communities that are attractive to livestock and thus are associated with higher livestock densities.

The ST zone appears to demonstrate characteristics of both equilibrium and non‐equilibrium dynamics (Fernandez‐Gimenez and Allen‐Diaz 1999), though recent changes in climate and grazing intensity could be pushing it toward the non‐equilibrium end of the continuum (Jamiyansharav et al. 2018, Jamsranjav et al. 2018). ST herders’ observations align with these findings, as they saw rainfall as the main cause of healthy rangelands, and perceived overgrazing as the main driver of degradation. In the ST, one herder also perceived irreversible change. In the ST, ordination analysis showed that plant community composition is associated most strongly with growing‐season precipitation and moderately associated with livestock use, similar to findings of Jamiyansharav et al. (2018). As in the DS, herders’ assessment scores correlated with the axis 1 and herders tended to give higher scores to *Stipa krylovii* dominated plots. As in the DS, ST herders may give higher scores to high quality pastures that attract livestock and thus have higher levels of livestock use.

Finally, rainfall in the ST was higher in the ecological sampling year (2012) than the interview year (2013), while in the DS, total precipitation was lower in the ecological sampling year (2011) than in the interview year (2013). The difference in precipitation between the ecological sampling and interview years is a limitation of our study. We would expect these differences to affect biomass and cover more than species composition, as most of the species at the study sites are perennial, and impacts to be greatest in the more variable DS. Yet herders’ assessment scores in the ST and DS correlated significantly with several of the measured indicators, and with the dominant NMS axes. Herder scores might have correlated even more strongly, or with more measured indicators, had sampling and interviews taken place in the same year. Because most of the plants are perennial, production, cover, and species composition along the long‐term grazing gradients we sampled represent longer‐term shifts in community structure and function more than interannual variability, especially in the ST and MFS. Nevertheless, we recommend that future studies using this approach conduct field sampling and interviews in the same year to maximize reliability.

### Management implications

**Figure 7:**
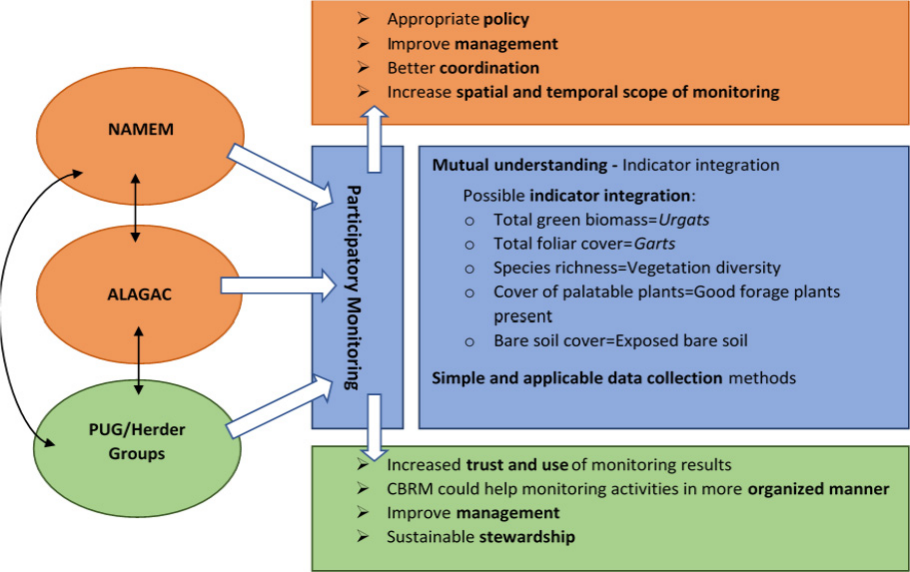
Proposed participatory monitoring, lists of possible integrated indicators and possible expected outcomes. Two government agencies conduct formal monitoring include NAMEM (National Agency for Meteorology and Environmental Monitoring) and ALAGAC (Administration of Land Affairs, Geodesy and Cartography). PUG, pasture user groups.

Given the large number of existing organized CBRM groups in Mongolia, we propose that herders be invited to engage more actively in formal monitoring, or to contribute their informal observations in a more systematic way to broad‐scale monitoring efforts. Fig. 7 proposes how this might work. Building upon a common terminology and set of indicators that are already used by both herders and government officials, herders could collect data using one of several methods. One option is to train herders to collect formal monitoring data using simple methods, as some CBRM projects have already done (Baival and Fernandez‐Gimenez 2012). However, this may require a greater time commitment than some herders are able to make, as well as access to monitoring tools and data forms, and a way to transmit the data to the appropriate government office. A second option is to use cell phones, which most herders already possess, and develop a simple interface where herders can record their quantitative assessment score on a simple 0–100 scale, and then document the observations they used to arrive at this score much as we did in our interviews. A 0–100 scale is appropriate in Mongolia as herders commonly think in terms of percentages. In addition to their observations, herders could attach photos of assessed pastures and specific features that informed their assessment, such as particular plants, plant communities or erosion indicators, for example. Several applications already exist with similar capabilities (e.g., LandPKS; Herrick et al. 2013, 2016) and PastureMap.^1^ For example, Land PKS is a mobile phone application where individual users provide their geo‐tagged estimates to a cloud‐based computing system to integrate, interpret, and create information and knowledge. Local people can access this system to exchange information and share their knowledge about land management with other locals. This approach would require minimal training, no tools other than a cell phone, and the data could be transmitted via the phone to a centralized database. It is particularly apt for the Mongolian context, where literacy rates are very high and cell phones and social media use are ubiquitous in the rural population.

Given the collective nature of land use and management in Mongolia, where private property is unconstitutional and herders in a given *soum* share pastures as commons, it is important that monitoring results be discussed, and management decisions taken at a group level. Although LandPKS allows individuals to access data and see their data in context, there is still a need for monitoring results to be communicated to and discussed among local CBRM groups in a timely fashion and understandable format to be used for group‐level pasture management decisions. This calls for some type of institutionalized herder/community involvement in monitoring at local, regional, and national levels (Danielsen et al. 2005).

We propose that if herders understand indicators used in formal monitoring and participate meaningfully in monitoring activities, they will trust the monitoring results and will be more likely to use them (Johnson et al. 2015). Thus, herders’ understanding of and, ideally, involvement in formal rangeland monitoring is key for improving rangeland management and sustainable stewardship. The institutional context in Mongolia is complex and there are many constraints on and factors influencing herders’ behavior and grazing management (Fernandez‐Gimenez and Batbuyan 2004, Addison and Brown 2014). Yet engaging herders in monitoring is likely a necessary if not sufficient condition to work toward more sustainable grazing management. Our experience and other published accounts (Baival and Fernandez‐Gimenez 2012) suggest that herders are interested in participating in monitoring, if it is simple for them to do. However, a more thorough feasibility assessment is needed before designing programs based on our findings. We recommend that a feasibility assessment involve herders who have been involved in past or current rangeland monitoring efforts associated with CBRM projects, as well as potential herder participants in future monitoring efforts, local government, and conservation organizations. The potential benefit of engaging existing CBRM organizations in this process is that they could help in all stages of monitoring, from data collection and data interpretation to making management decisions. CBRMs could coordinate local herder monitoring efforts in an organized manner (perhaps in national herder monitoring days each year, for example), facilitate community‐level discussions about monitoring results and their interpretation, and serve as a communication link between local government and herders. Perhaps most critically, as CBRM groups have a growing voice in managing rangelands and enforcing management rules among members, they may play a key role in using monitoring data to make and enforce local management decisions.

There are multiple potential benefits to government agencies of institutionalizing herder/community involvement in rangeland monitoring. Potential benefits to government include (1) increased spatial and temporal scope of data collection; (2) improved coordination among local, regional and national monitoring agencies; (3) improved national‐level understanding of the health of rangelands and hotspots needing management attention; (4) increased buy‐in by herders for the need for specific management interventions; (5) more robust national discussion on the most needed rangeland policies and interventions; and (6) more appropriate policies, especially policies regulating movements between *soums* during droughts or other forage emergencies (Fernandez‐Gimenez et al. 2012). We emphasize that these hypothesized benefits represent the promise of greater herder involvement in formal monitoring. As with any policy shift, we advocate a rigorous evaluation and learning component to assess whether or to what degree these benefits are realized.

Finally, we suggest that the use of a mobile app could help to engage young herders and Mongolian youth generally in learning, applying, and thus conserving traditional herder knowledge. The future of traditional knowledge in Mongolia is potentially threatened by rural to urban migration (Fernandez‐Gimenez et al. 2017), and a declining and aging herder population. Our proposed app‐based participatory rangeland monitoring approach could engage young herders in monitoring and encourage urban migrants to access monitoring results for their homelands, assessed by their parents and other herders. We speculate that this approach could stimulate discussion between youth and older herders about rangeland conditions. Further, it may provide an opportunity to transfer TEK and specific place‐based knowledge to young generations who have migrated to urban areas, even if they do not use rangelands actively. This transfer would occur via the app, where senior herders, family and community members, relay their observations and knowledge, and younger urban Mongolians retrieve them. Although we do not see the app as a substitute for face‐to‐face interactions with elders and direct experience on the land, it could help to maintain youth interest, engagement, and traditional knowledge as pastoral culture adapts and evolves in the digital era.

In conclusion, herders’ ecological knowledge is rich and comprehensive, often expressed in qualitative form. Researchers have been attempting to integrate TEK and scientific knowledge to develop integrated indicators for participatory rangeland monitoring (Stringer and Reed 2007, Roba and Oba 2009, Raymond et al. 2010). Continuing challenges include quantifying herders’ knowledge (Knapp and Fernandez‐Gimenez 2009) for developing integrated indicators, finding simple and easy tools for herders to use for participatory rangeland monitoring, and developing systems that provide quick and timely results to local users. Our study demonstrates the potential for herders to provide simple quantitative ratings accompanied by supporting qualitative observations that both herders and monitoring specialists could use in participatory monitoring to detect changes in rangeland condition.

Our results and above recommendations are tailored to the specific ecological, management, and policy contexts of Mongolia. However, the methods we used to document herder TEK and analyze it quantitatively in relation to researcher‐measured rangeland monitoring indicators have broad applicability to rangeland and pastoral systems globally. Similarly, the solutions we recommend for Mongolia can be adapted to other contexts. In most rangelands globally, herders’ participation in rangeland monitoring and evaluation remains low, although there is increasing recognition of the potential contributions of local communities to these endeavors. As this study demonstrates, herders’ daily observations have the potential to play an important role in participatory monitoring. Comparing and combining assessments conducted by herders and formal monitoring and developing key indicators that both herders and government rangeland monitoring officials understand and interpret in same way, are important initial steps to engage herders in participatory monitoring. This process contributes to integrating existing informal herder monitoring with present and future formal monitoring and evaluation. The use of widely available contemporary technologies such as mobile phone applications is a promising approach to engaging herders in participatory monitoring, and potentially to maintaining knowledge transfer between rural elders and increasingly urban youth. Such app‐based tools also potentially reduce the time and cost of monitoring data collection. However, we suggest that face‐to‐face community discussions are needed to collectively discuss and apply monitoring results to community‐level management decisions. This combination of crowd sourced knowledge from cell‐phone apps, grounded in combined, mutually understood indicators, and community discussion could support meaningful local participation in monitoring and management, as called for in the UNCCD strategic plan (UNCCD 2015, Cowie et al. 2018) other international conventions.

## Data Availability

Data are available from the Dryad Digital Repository: https://doi.org/10.5061/dryad.bh0pj76

## Note

1 https://pasturemap.com/
