# Supplementary material for: Opportunities to integrate herders’ indicators into formal rangeland monitoring: an example from Mongolia
Source: Ecol Appl. 2019 May 17;29(5):e01899. doi: 10.1002/eap.1899 (PMC6851969; doi:10.1002/eap.1899)
Supplement: Supplementary file 1 [file EAP-29-na-s001.pdf]

Chantsallkham Jamsranjav, María E. Fernández-Giménez, Robin S. Reid, and B. Adya. 2019. Opportunities to integrate herders' indicators into formal rangeland monitoring: An example from Mongolia. *Ecological Applications.*

APPENDIX S1.

Questionnaire No____

INTERVIEW 1: INDIVIDUAL HERDERS' OBSERVATION

HERDERS' OBSERVATIONS OF RANGELAND CONDITIONS AND CHANGES AT THEIR WINTER CAMP AREA (UVULJUU)

Hello. My name is ____________ and we are from the Colorado State University. Our team's research topic is herder's knowledge of rangelands and their experience of its ecological change. As you may remember that our social study team conducted a household survey in 2010/2011 and we came and measured grass and soils near your uvuljuu in 2011/2012. The analysis part of this research is in progress. We will share some preliminary results of the analysis at the end of our interview. Today, in this interview, we are asking for your observations of your rangeland and the changes you've seen. We are interested in your thoughts and opinions, and there is no right or wrong answer to the questions. Our discussion takes an hour and half to complete. Before starting, do you have any questions? If you have questions during the interview, you should feel free to ask. In order to document your comments, is it OK if I record this interview? Thank you! (Remember to give gifts and dzud books now; research results at end)

Date:_____________Time at start:____________Time at end:________________________

Location (GPS) N:____________ E: ___________________________
Which ……_________________
Aimag ____________________
Soum_____________________
Bag______________
Uvuljuu name_______________
CBNRM group: YES____NO____
If *YES,* CBNRM group name _____________________________
If *NO,* Bag/valley(neighborhood) name ________________________

I.A. General information about household

1. Respondent's full name: ___________________________________
2. Age: ___________________________________
3. Sex: Male _____ Female______

I.B. History of land use at this uvuljuu (settlement, livestock numbers and types, other uses)

1. In what year did you start using this uvuljuu (winter camp)? ______

2. What year was this uvuljuu first used?
________year ___I do not know. If they do not know, how many years has the uvuljuu been used?

3. Have you used your uvuljuu every year since you first started using it?
_____YES
_____ NO. If *NO,* then ask: In the last 10 years, when did you not use the uvuljuu? And when you did not use the uvuljuu, what was the reason did you not use the uvuljuu? Did someone else use it then or was it empty during the winter?

Year	Reason we did not use the uvuljuu	Was someone else here that year? (yes, no, don't know)

4. How many families stayed together in this uvuljuu over the last 3 winters?

Year, winter	# of families who stayed together here
2010-2011 winter	
2011-2012 winter	
2012-2013 winter	

5. How many livestock of what types did you have at the uvuljuu (of all families living at the uvuljuu) during the winter of the following three time periods?

Years	Camel	Horse	Cattle (Put # of Yak in parenthesis	Sheep	Goat	Months stayed at the uvuljuu
2012-2013						
2011-2012						
2010-2011						
2000-2001						
1993-1994						

6. Do you or others use the grazing lands around the uvuljuu in other seasons?
   a. YES **b.**NO

7. If *YES*, for the last two years, approximately how many and what types of livestock were grazed

here, in what season(s) and for how long? Types of
livestock____
Seasons used____
Which months used each year____

Livestock type	Camel	Horse	Cattle	Sheep	Goat
Number					

8. If *YES*, how far away from the uvuljuu do you collect dung? And what species of dung did you collect?
   ___direction from the uvuljuu
   ___distance from uvuljuu (m)

II. Definition of Rangeland Health

To the interviewer: lay out stick of a fixed length (a meter stick, for example) with four at least equal length marks at ¼, ½ and ¾ across the stick. Then use this tool to ask the following questions.

1. When you look at your grazing areas, what aspects (or indicators) of that grazing area tell you it is healthy and in good condition for livestock? (list)

2. What words do you use to describe a healthy grazing area and how do they differ in meaning (ask for examples if it is not clear)?

3. What are your indicators of an unhealthy (degraded) rangeland?

4. What words do you use to describe an unhealthy grazing area and how do they differ in meaning (ask for examples if it is not clear)

5. What causes a rangeland to be healthy? What causes a rangeland to become unhealthy? (write down causes in respective column)

Causes of health or degradation	Healthy	Unhealthy (degraded)

6. Which of these causes of rangeland health has the greatest impact on the condition of your pastures?

Causes	Ranking of causes

III. Plot Vegetation Condition at the winter camp (uvuljuu)
Instruction to interviewer: Go to the first 50 x 50 m plot and place flagged pins at the four corners of the plots based on the GPS points. Then walk with the herder in a zigzag way through the entire plot as you

ask the following questions:

QUESTIONS 1.1 - 1.13 BELOW SHOULD BE ASKED AT THE 100m PLOT

1. 1. Where would you place the OVERALL condition of this plot on the stick / line from a very healthy pasture to a very unhealthy pasture? (Record here where they point to on the stick)? .

0 cm = Most unhealthy, 40cm = Most healthy

1.2. For this plot, remembering the indicators (signs) you listed above, what are the indicators (signs)are you using to judge the condition of this plot? Of these indicators (signs), which one is most useful in ranking the condition of the pasture in the plot against each of your indicators?

Indicators	Rank (most useful-5, least useful-1

1.3. Based on the plants growing in the plot, how would you rate the quality of the forage in this plot for your different kinds of livestock (use the stick again)?

Camels	Horses	Cattle/Yak	Sheep	Goats

1.4 Any observations about the condition of the soils in this plot?

1.5 Other observations the herders mention about the plot:

1.5+ Do you collect dung in this plot?

1.6 You said the plot was in _____ condition, what factors explain why this plot is in this condition? (List the factors and once they are done with their list, and then ask them to rank the importance of these factors from most to least important and ask causes of factors.

Factors explaining the current <u>conditions</u>	Rank importance (5-most 1-least)	Causes and any other notes
1.		
2.		

3.		
4.		
5.		

1.7. Could the condition of the vegetation and soils in this plot be improved or is it impossible to improve?

a. YES, it can be improved **b**.NO, it cannot be improved

1.8. If *YES*, what could improve the condition of this plot? (examples: management practices, weather)

1.9. If *YES*, how long will it take to recover this plot if these things happened? ________(years)

1.10. If NO, why not?

QUESTIONS 2.1 - 2.13 BELOW SHOULD BE ASKED AT 500m PLOT

2.1. Where would you place the OVERALL condition of this plot on the stick / line from a very healthy pasture to a very unhealthy pasture? (Record here where they point to on the stick)? .

0 cm = Most unhealthy, 40cm = Most healthy

2.2. For this plot, remembering the indicators (signs) you listed above, what are the indicators (signs)are you using to judge the condition of this plot? Of these indicators (signs), which one is most useful in ranking the condition of the pasture in the plot against each of your indicators?

Indicators	Rank (most useful-5, least useful-1

2.3. Based on the plants growing in the plot, how would you rate the quality of the forage in this plot for your different kinds of livestock (use the stick again)?

Camels	Horses	Cattle/Yak	Sheep	Goats

2.4. Any observations about the condition of the soils in this plot?

2.5. Other observations the herders mention about the plot:

2.5+ Do you collect dung from this plot?

2.6. You said the plot was in _____ condition, what factors explain why this plot is in this condition? (List the factors and once they are done with their list, and then ask them to rank the importance of these factors from most to least important and ask causes of factors.

Factors explaining the current conditions	Rank importance (5-most 1-least)	Causes and any other notes
1.		
2.		
3.		
4.		
5.		

2.7. Could the condition of the vegetation and soils in this plot be improved or is it impossible to improve?

a. YES, it can be improved **b**.NO, it cannot be improved

2.8. If *YES*, what could improve the condition of this plot? (examples: management practices, weather)

2.9. If *YES*, how long will it take to recover this plot if these things happened? ________(years)

2.10. If NO, why not?

QUESTIONS 3.1 - 3.13 BELOW SHOULD BE ASKED AT THE 1000m PLOT

3.1. Where would you place the OVERALL condition of this plot on the stick / line from a very healthy pasture to a very unhealthy pasture? (Record here where they point to on the stick)? .

0 cm = Most unhealthy, 40cm = Most healthy

3.2. For this plot, remembering the indicators (signs) you listed above, what are the indicators (signs)are you using to judge the condition of this plot? Of these indicators (signs), which one is most useful in ranking the condition of the pasture in the plot against each of your indicators?

Indicators	Rank (most useful-5, least useful-1

3.3. Based on the plants growing in the plot, how would you rate the quality of the forage in this plot for your different kinds of livestock (use the stick again)?

Camels	Horses	Cattle/Yak	Sheep	Goats

3.4. Any observations about the condition of the soils in this plot?

3.5. Other observations the herders mention about the plot:

3.5+ Do you collect dung from this plot?

3.6. You said the plot was in _____ condition, what factors explain why this plot is in this condition? (List the factors and once they are done with their list, and then ask them to rank the importance of these factors from most to least important and ask causes of factors.

Factors explaining the current <u>conditions</u>	Rank importance (5-most 1-least)	Causes and any other notes
1.		
2.		

3.		
4.		
5.		

3.7. Could the condition of the vegetation and soils in this plot be improved or is it impossible to improve?

a. YES, it can be improved **b**.NO, it cannot be improved

3.8. If *YES*, what could improve the condition of this plot? (examples: management practices, weather)

3.9. If *YES*, how long will it take to recover this plot if these things happened? ________(years)

3.10. If NO, why not?

APPROVED

Thank you very much for your thoughtful responses. Thank you very much for your time.
