# Supplementary material for: Opportunities to integrate herders’ indicators into formal rangeland monitoring: an example from Mongolia
Source: Ecol Appl. 2019 May 17;29(5):e01899. doi: 10.1002/eap.1899 (PMC6851969; doi:10.1002/eap.1899)
Supplement: Supplementary file 2 [file EAP-29-na-s002.pdf]

Chantsallkham Jamsranjav, María E. Fernández-Giménez, Robin S. Reid, and B. Adya. 2019. Opportunities to integrate herders' indicators into formal rangeland monitoring: An example from Mongolia. *Ecological Applications.*

APPROVED

APPENDIX S2.

Table S1. Summary of plant species mean absolute cover and frequency in each community group in the mountain and forest steppe (MFS) ecological zone.

**Species name (code)**	**Community Group 1 (n=12)** *Cleistogenes squarrosa/Artemisia frigida/Carex duriuscula*		**Community Group 2 (n=4)** *Poa attenuata/Carex korshinskyi*		**Community Group 3 (n=8)** *Agropyron cristatum/Allium senescens/Caragana microphylla*	
**Perennial Grasses**	**Cover (%)**	**Frequency**	**Cover (%)**	**Frequency**	**Cover (%)**	**Frequency**
Agropyron cristatum (AGCR)	1.60	34	0.10	2	3.00	64
Alopecurus arundunaceus (ALAR)	0	0	0.10	100	0	0
Cleistogenes squarrosa (CLSQ)	9.60	72	0	0	3.72	28
Elymus chinensis (ELCHN)	1.30	12	4.6	43	4.8	45
Festuca lenensis (FELE)	0.03	6	0.50	94	0	0
Koeleria macrantha (KOMA)	0.83	40	0.75	36	0.50	24
Poa attenuate (POAT)	0	0	17.9	91	1.68	9
Stipa krylovii (STKR)	14.95	21	17.50	25	38.20	54
Stipa sibirica (STSB)	0.10	20	0	0	0.40	80
**Annual Grasses**	**Cover (%)**	**Frequency**	**Cover (%)**	**Frequency**	**Cover (%)**	**Frequency**
Hordeum vulgare (HOVU)	0	0	1.60	100	0	0
**Sedges**	**Cover (%)**	**Frequency**	**Cover (%)**	**Frequency**	**Cover (%)**	**Frequency**
Carex duriuscula (CXDU)	13.53	46	6.10	21	9.65	33
Carex Korshinskyi (CXKO)	0.43	5	8.45	92	0.30	3
**Perennial Forbs**	**Cover (%)**	**Frequency**	**Cover (%)**	**Frequency**	**Cover (%)**	**Frequency**

Allium anisopodium (ALLAN)	0	0	0	0	0.05	100
Allium bidentatum (ALLBI)	0	0	0.10	40	0.15	60
Allium lineare (ALLLI)	0.07	100	0	0	0	0
Allium mongolicum (ALLMGL)	0	0	0	0	0.05	100
Allium senescens (ALLSE)	0.03	2	0	0	1.55	100
Arenaria capillaris (ARCA)	0.43	100	0	0	0	0
Arctogeron gramineum (ARGR)	0.03	100	0	0	0	0
Artemisia pamirica (ARPA)	0.03	100	0	0	0	0
Astragalus galactites (ASGA)	0.10	40	0.10	40	0.05	20
Bupleurum bicaule (BUBI)	0	0	0.60	100	0	0
Convolvulus Ammanii (COAM)	0.17	53	0.1	32	0.05	16
Cymbaria dahurica (CYDH)	0.33	34	0.20	20	0.45	46
Dianthus versicolor (DIVE)	0.40	13	2.50	78	0.30	9
Echinops humilis (ECHU)	0.03	100	0	0	0	0
Galium verum (GAVE)	0.93	41	0.90	39	0.45	20
Geranium dahuricum (GERDH)	0	0	0.30	100	0	0
Haplophyllum dauricum (HADA)	0	0	0.10	100	0	0
Iris tigrida (IRTI)	0.07	100	0	0	0	0
Leontopodium ochroleucum (LEOC)	0	0	0.3	100	0	0
Libanotis buchtormensis (LIBU)	0	0	0.20	100	0	0
Medicago falcata (MEFA)	0	0	0.20	100	0	0
Pedicularis flava (PEFL)	0.13	100	0	0	0	0
Phlomis tuberosa (PHTU)	0	0	0.50	100	0	0
Polygonum angustifolium (PLAN)	0.03	100	0	0	0	0

Polygonum divaricatum (PLDI)	0.03	100	0	0	0	0
Potentilla acaulis (POTAC)	3.33	61	0.60	11	1.50	28
Potentilla bifurca (POTBI)	0.33	57	0.20	34	0.05	9
Potentilla leucophylla (POTLE)	0.30	75	0.10	25	0	0
Potentilla multifida (POTMU)	0	0	1	100	0	0
Potentilla tanacetifolia (POTTA)	0.03	4	0.80	96	0	0
Ptilotrichum canescens (PTCA)	0	0	0.10	100	0	0
Pulsatilla ambigua (PUAM)	0.77	34	0.80	36	0.68	30
Saposhnikovia divaricata (SADI)	0.17	53	0	0	0.15	47
Saussurea salicifoli (SASA)	0.07	7	0.9	93	0	0
Scorzonera austriaca (SCAU)	0.03	40	0	0	0.05	60
Serratula centauroides (SECE)	0	0	0	0	0.05	100
Sibbaldianthe adpressa (SIAD)	0.03	18	0	0	0.15	82
Stellera chamaejasme (STCHA)	0.03	100	0	0	0	0
Stellaria dichotoma (STDI)	0.03	100	0	0	0	0
Thermopsis dahurica (THDAH)	0	0	0.60	100	0	0
Thalictrum simplex (THIS)	0	0	1.30	93	0.10	70
Veronica incana (VEIN)	0.13	28	0.2	41	0.15	31
Vicia cracca (VICR)	0.03	1	3.10	99	0	0
**Annual forbs**	**Cover (%)**	**Frequency**	**Cover (%)**	**Frequency**	**Cover (%)**	**Frequency**
Artemisia palustris (ARPAL)	0.33	77	0.10	23	0	0
Artemisia Sieversiana (ARSI)	0	0	0	0	0.15	100
Chenopodium album (CHAL)	0	0	0	0	0.50	100
Chamaerhodos erecta (CHERE)	0.03	100	0	0	0	0

Dontostemon integrifolius (DOIN)	0.03	14	0	0	0.2	86
Heteropappus hispidus (HEHI)	1.00	30	0	0	2.35	70
Lappula myosotis (LAMY)	0.17	100	0	0	0	0
Lappula stricta (LAST)	0.03	100	0	0	0	0
**Subshrubs and Shrubs**	**Cover (%)**	**Frequency**	**Cover (%)**	**Frequency**	**Cover (%)**	**Frequency**
Artemisia adamsii (ARAD)	1.47	57	1.10	43	0	0
Artemisia commutata (ARCO)	0	0	2.0	88	0.40	12
Artemisia frigida (ARFRI)	8.83	77	0.40	3	2.20	19
Kochia prostrata (KOPR)	1.63	48	0.50	15	1.30	38
Caragana micropylla (CARMI)	0.53	20	0.10	4	2.05	76
Caragana pygmaea (CARPY)	0.73	44	0.10	6	0.85	50
Caragana stenophylla (CARST)	0	0	0	0	0.15	100
