# Supplementary material for: Opportunities to integrate herders’ indicators into formal rangeland monitoring: an example from Mongolia
Source: Ecol Appl. 2019 May 17;29(5):e01899. doi: 10.1002/eap.1899 (PMC6851969; doi:10.1002/eap.1899)
Supplement: Supplementary file 3 [file EAP-29-na-s003.pdf]

Chantsallkham Jamsranjav, María E. Fernández-Giménez, Robin S. Reid, and B. Adya. 2019. Opportunities to integrate herders' indicators into formal rangeland monitoring: An example from Mongolia. *Ecological Applications.*

APPROVED

APPENDIX S3.

Table S1. Summary of plant species mean absolute cover and frequency in each community group in the steppe (ST) ecological zone.

**Species**	**Community Group 1 (n=10)** *Cleistogenes squarrosa/ Carex duriuscula Allium polyrrhizum/ Artemisia frigida*		**Community Group 2 (n=9)** *Stipa krylovii*		**Community Group 3 (n=3)** *Agropyron cristatum/ Artemisia adamsii/ Chenopodium album*		**Community Group 4 (n=3)** *Stipa gobica/ Kochia prostrata*		**Community Group 5 (n=2)** *Elymus chinensis*	
**Perennial Grasses**	**Cover (%)**	**Frequency**	**Cover (%)**	**Frequency**	**Cover (%)**	**Frequency**	**Cover (%)**	**Frequency**	**Cover (%)**	**Frequency**
Achnatherum splendens (ACSP)	0.08	6	0	0	1.20	94	0	0	0	0
Agropyron cristatum (AGCR)	3.48	20	3.78	21	8.40	47	1.9	11	0.20	1
Cleistogenes squarrosa (CLSQ)	8.40	53	1.73	11	3.1	19	1.1	7	1.6	10
Elymus chinensis (ELCHN)	6.64	13	1.64	3	0.50	1	0	0	40.4	82
Koeleria glauca (KOGL)	0.36	38	0.18	19	0.10	14	0.30	28	0	0
Koeleria macrantha (KOMA)	0.04	100	0	0	0	0	0	0	0	0
Stipa krylovii (STKR)	32.64	26	59.38	48	5.80	5	13.7	11	12.2	10
Stipa gobica (STGB)	0.36	1	7.91	16	0	0	42.1	84	0	0

**Sedges**										
Carex duriuscula (CXDU)	10.80	55	0.80	4	3.60	18	0.10	1	4.20	22
Carex Korshinskyi (CXKO)	0	0	0.04	100	0	0	0	0	0	0
**Perennial Forbs**	**Cover (%)**	**Frequency**	**Cover (%)**	**Frequency**	**Cover (%)**	**Frequency**	**Cover (%)**	**Frequency**	**Cover (%)**	**Frequency**
Allium anisopodium (ALLAN)	0.96	11	2.76	30	1.3	15	4.00	44	0	0
Allium bidentatum (ALLBI)	0.20	100	0	0	0	0	0	0	0	0
Allium polyrrhizum (ALLPO)	4.68	57	2.00	24	1.60	19	0	0	0	0
Allium senescens (ALLSE)	0	0	0.40	50	0.10	17	0.30	33	0	0
Arenaria capillaris (ARCA)	0.08	37	0.13	63	0	0	0	0	0	0
Artemisia dracunculus (ARDR)	0.40	50	0	0	0	0	0	0	0.40	50
Bupleurum bicaule (BUBI)	0.12	47	0.13	53	0	0	0	0	0	0
Convolvulus Ammanii (COAM)	1.32	16	3.11	37	0	0	4.00	47	0	0
Cymbaria dahurica (CYDH)	0.44	32	0.13	10	0	0	0.80	58	0	0
Dianthus soongoricus (DISO)	0	0	0.04	100	0	0	0	0	0	0
Dianthus versicolor (DIVE)	0.08	100	0	0	0	0	0	0	0	0
Ephedra equisetina (EPEQ)	0.08	37	0	0	0.10	63	0	0	0	0

Galium verum (GAVE)	0.16	100	0	0	0	0	0	0	0	0
Haplophyllum dauricum (HADA)	0.04	31	0.09	69	0	0	0	0	0	0
Iris tigrida (IRTI)	0.08	31	0.18	69	0	0	0	0	0	0
Oxytropis filiformus (OXFI)	0.24	47	0	0	0.30	53	0	0	0	0
Pedicularis dasystachys (PEDA)	0	0	0	0	0	0	0	0	0.20	100
Potentilla acaulis (POTAC)	0.04	47	0.04	53	0	0	0	0	0	0
Potentilla bifurca (POTBI)	0	0	0.04	100	0	0	0	0	0	0
Potentilla leucophylla (POTLE)	0	0	0.04	100	0	0	0	0	0	0
Potentilla sericea (POTSE)	0	0	0.04	100	0	0	0	0	0	0
Potentilla tanacetifolia (POTTA)	0.04	23	0	0	0	0	0.10	77	0	0
Pulsatilla ambigua (PUAM)	0.16	16	0.13	13	0.10	13	0	0	0.60	58
Saposhnikovia divaricata (SADI)	0.08	31	0.18	69	0	0	0	0	0	0
Saussurea salicifoli (SASA)	0	0	0.13	100	0	0	0	0	0	0
Serratula centauroides (SECE)	0.12	18	0.13	20	0.40	61	0	0	0	0
Thalictrum simplex (THIS)	0.04	100	0	0	0	0	0	0	0	0
Trifolium lupinaster (TRLU)	0.16	55	0	0	0.10	45	0	0	0	0

**Annual forbs**	**Cover (%)**	**Frequency**	**Cover (%)**	**Frequency**	**Cover (%)**	**Frequency**	**Cover (%)**	**Frequency**	**Cover (%)**	**Frequency**
Artemisia mongolica (ARMGL)	0.08	64	0.04	36	0	0	0	0	0	0
Chenopodium album (CHAL)	0.08	4	0.40	20	1.30	66	0	0	0.20	10
Corispermum mongolicum (COMGL)	0.12	100	0	0	0	0	0	0	0	0
Dontostemon integrifolius (DOIN)	0.04	100	0	0	0	0	0	0	0	0
Heteropappus hispidus (HEHI)	0.40	50	0	0	0.40	50	0	0	0	0
Polygonum dumetorum (PLDU)	0	0	0.31	100	0	0	0	0	0	0
**Subshrubs and Shrubs**	**Cover (%)**	**Frequency**	**Cover (%)**	**Frequency**	**Cover (%)**	**Frequency**	**Cover (%)**	**Frequency**	**Cover (%)**	**Frequency**
Artemisia adamsii (ARAD)	2.92	57	0.22	4	2.00	39	0	0	0	0
Artemisia frigida (ARFRI)	6.32	25	1.16	5	3.7	15	0	0	13.6	55
Asparagus gobicus (ASGO)	0	0	0.04	100	0	0	0	0	0	0
Atriplex cana (ATCA)	0.24	100	0	0	0	0	0	0	0	0
Kochia prostrata (KOPR)	1.36	8	2.18	14	2.5	16	5.6	35	4.4	27
Thymus gobicus (THGO)	0.04	100	0	0	0	0	0	0	0	0
Caragana micropylla (CARMI)	0.16	78	0.04	22	0	0	0	0	0	0

Caragana stenophylla (CARST)	2.36	31	0.89	12	2.00	26	0.90	12	1.40	18
