# Supplementary material for: Opportunities to integrate herders’ indicators into formal rangeland monitoring: an example from Mongolia
Source: Ecol Appl. 2019 May 17;29(5):e01899. doi: 10.1002/eap.1899 (PMC6851969; doi:10.1002/eap.1899)
Supplement: Supplementary file 4 [file EAP-29-na-s004.pdf]

APPROVED

Chantsallkham Jamsranjav, María E. Fernández-Giménez, Robin S. Reid, and B. Adya. 2019. Opportunities to integrate herders' indicators into formal rangeland monitoring: An example from Mongolia. *Ecological Applications.*

APPENDIX S4.

Table S1. Summary of plant species mean absolute cover and frequency in each community group in the desert steppe (DS) ecological zone.

**Species**	**Community Group 1 (n=15)** *Allium mongolicum/ Salsola collina*		**Community Group 2 (n=4)** *Allium polyrrhizum/ Caragana stenophylla*		**Community Group 3 (n=5)** *Stipa gobica/ Allium mongolicum*		**Community Group 4 (n=3)** *Eragrostis minor/ Dontostemon integrifolius*	
**Perennial Grasses**	**Cover (%)**	**Frequency**	**Cover (%)**	**Frequency**	**Cover (%)**	**Frequency**	**Cover (%)**	**Frequency**
Achnatherum splendens (ACSP)	0.05	100	0	0	0	0	0	0
Cleistogenes squarrosa (CLSQ)	0.91	32	0.50	18	0.64	22	0.80	28
Stipa gobica (STGB)	3.47	9	7.9	21	21.28	58	4.13	11
**Annual Grasses**	**Cover (%)**	**Frequency**	**Cover (%)**	**Frequency**	**Cover (%)**	**Frequency**	**Cover (%)**	**Frequency**
Eragrostis minor (ERMI)	0.51	5	0.60	6	0.40	4	8.80	85
**Sedges**	**Cover (%)**	**Frequency**	**Cover (%)**	**Frequency**	**Cover (%)**	**Frequency**	**Cover (%)**	**Frequency**
Carex duriuscula (CXDU)	0	0	0.10	100	0	0	0	0
Carex pediformis (CXPED)	0.29	23	0.50	39	0.08	6	0.40	31
**Perennial Forbs**	**Cover (%)**	**Frequency**	**Cover (%)**	**Frequency**	**Cover (%)**	**Frequency**	**Cover (%)**	**Frequency**

Allium anisopodium (ALLAN)	0	0	0.3	100	0	0	0	0
Allium mongolicum (ALLMGL)	2.51	28	0.9	10	0	0	0	0
Allium polyrrhizum (ALLPO)	2.24	13	14.1	81	0.88	5	0.27	2
Artemisia dracunculus (ARDR)	0	0	0	0	0	0	0.13	100
Convolvulus ammanii (COAM)	0.51	36	0.60	43	0.16	11	0.13	10
Gypsophila desertorum (GYDE)	0.03	17	0	0	0	0	0.13	83
Peucedanum hystrix (PEHY)	0	0	0	0	0	0	0.40	100
Peganum nigellastrum (PENI)	0.08	100	0	0	0	0	0	0
Scorzonera pseudodivaricata (SCPS)	0	0	0.20	14	0	0	1.20	86
**Annual forbs**	**Cover (%)**	**Frequency**	**Cover (%)**	**Frequency**	**Cover (%)**	**Frequency**	**Cover (%)**	**Frequency**
Bassia dasyphylla (BADA)	0.08	38	0	0	0	0	0.13	63
Chenopodium album (CHAL)	0.19	27	0.1	15	0	0	0.40	58
Dontostemon integrifolius (DOIN)	0.45	5	0	0	0.32	4	7.87	91
Dontostemon senilis (DOSE)	0.05	18	0	0	0.24	82	0	0
Halogeton glomeratus (HAGL)	0	0	0	0	0	0	0.53	100
Heteropappus hispidus (HEHI)	0	0	0	0	0.08	100	0	0
Salsola collina (SACOL)	2.32	47	0.10	2	0.08	2	2.47	50
Setaria viridis (SEVI)	0	0	0	0	0	0	0.27	100

Tribulus terrestris (TRTE)	0.03	6	0	0	0	0	0.40	94
**Subshrubs and Shrubs**	**Cover (%)**	**Frequency**	**Cover (%)**	**Frequency**	**Cover (%)**	**Frequency**	**Cover (%)**	**Frequency**
Ajania achileoides (AJAC)	0.21	68	0.10	32	0	0	0	0
Ajania fruticulosa (AJFR)	0	0	0	0	0.08	23	0.27	77
Anabasis brevifolia (ANBR)	0.32	100	0	0	0	0	0	0
Artemisia rutifolia (ARRUT)	0	0	0	0	0.16	100	0	0
Artemisia xerophytica (ARXE)	0.19	100	0	0	0	0	0	0
Asterothamnus alyssoides (ASALY)	0	0	0	0	0	0	0.13	100
Asparagus gobicus (ASGO)	0.13	8	0.2	12	0	0	1.33	80
Eurotia ceratiodes (EUCE)	0.05	14	0	0	0.32	86	0	0
Caragana stenophylla (CARST)	0.19	15	0.80	65	0.24	20	0	0
Oxytropis aciphylla (OXACI)	0	0	0	0	0	0	0.13	100
Salsola passerina (SAPA)	0.32	55	0	0	0	0	0.27	45
Zygophyllum xanthoxylon (ZYXA)	0.05	100	0	0	0	0	0	0
