# Supplementary material for: Opportunities to integrate herders’ indicators into formal rangeland monitoring: an example from Mongolia
Source: Ecol Appl. 2019 May 17;29(5):e01899. doi: 10.1002/eap.1899 (PMC6851969; doi:10.1002/eap.1899)
Supplement: Supplementary file 5 [file EAP-29-na-s005.pdf]

Chantsallkham Jamsranjav, María E. Fernández-Giménez, Robin S. Reid, and B. Adya. 2019. Opportunities to integrate herders' indicators into formal rangeland monitoring: An example from Mongolia. *Ecological Applications.*

APPROVED

APPENDIX S5.

Table S1. Summary of selected vegetation, climate and environmental characteristics of community types in the mountain and forest steppe (MFS) ecological zone. Values are means ± standard error (SE). Community groups were compared for differences in plant functional type cover and biomass, litter biomass and species richness using ANOVA and a Tukey-adjusted multiple comparison test. Groups that differed from each other ($P < 0.05$) are indicated with different superscripts.

**Variables**	**Community Group 1 (n=12)** *Cleistogenes squarrosa/Artemisia frigida/Carex duriuscula*	**Community Group 2 (n=4)** *Poa attenuata/Carex korshinskyi*	**Community Group 3 (n=8)** *Agropyron cristatum/Allium senescens/Caragana microphylla*
Grass cover (%)	32.2±2.6	45.3±7.1	54.6±4.6
Forb cover (%)	9.7±1.8	15.9±2.4	10.4±1.8
Sedge cover (%)	14±2.5	16.8±1.5	10±2.8
Shrub cover (%)	1.3±0.3	5.1±5	3±0.5
Subshrub cover (%)	12±1.4	4.9±1.3	3.9±1.3
Palatable plant cover (%)	59.9±3.2[a]	72.2±9.2[ab]	75.2±3.9[b]
Unpalatable plant cover (%)	9.3±1.9	15.3±5.1	6.8±1.9
Annual plant cover (%)	1.6±0.8	1.7±0.9	3.2±1
Perennial plant cover (%)	67.5±3.1	86.3±3.9	78.7±3.2
Total foliar cover (%)	69.1±2.8	88±4.3	81.9±2.9
Grass biomass (g/m^2)	26.8±3.6[a]	23.4±2.8[a]	50.9±4.2[b]
Forb biomass (g/m^2)	37.9±3.4	48.8±9	32.8±5.3
Sedge biomass (g/m^2)	8.3±1.5	6.8±0.9	4.9±2.2
Shrub biomass (g/m^2)	19.3±5.2	0.8±0.5	17±4.9
Total green biomass (g/m^2)	92.3±8.7	79.8±11.2	105.6±9
Litter biomass (g/m^2)	34.4±5.4	23.6±1.6	29.3±8.9
Species richness	38±2.4[b]	54.3±2.9[a]	33.3±1.9[b]

Mean growing season precipitation (mm)	314±2	318.9±0	305.2±0
Mean annual precipitation (mm)	281.1±0.3	281.9±0	279.5±0
Mean annual temperature (ºC)	-1.1±0.03	-1.2±0.1	-1.1±0.05
Aspect	194.5±34.5	114.5±62.8	183.1±46.9
Elevation (m)	922.2±11.6	1028±48.4	904.2±13.5
Slope (degrees)	8.1±1.3	16.2±6.3	7.3±1.7
