# Supplementary material for: Opportunities to integrate herders’ indicators into formal rangeland monitoring: an example from Mongolia
Source: Ecol Appl. 2019 May 17;29(5):e01899. doi: 10.1002/eap.1899 (PMC6851969; doi:10.1002/eap.1899)
Supplement: Supplementary file 6 [file EAP-29-na-s006.pdf]

Chantsallkham Jamsranjav, María E. Fernández-Giménez, Robin S. Reid, and B. Adya. 2019. Opportunities to integrate herders' indicators into formal rangeland monitoring: An example from Mongolia. *Ecological Applications.*

APPROVED

APPENDIX S6.

Table S1. Summary of selected vegetation, climate and environmental characteristics of community types in the steppe (ST) ecological zone. Values are means ± standard error (SE). Community groups were compared for differences in plant functional type cover and biomass, litter biomass and species richness using ANOVA and a Tukey-adjusted multiple comparison test. Groups that differed from each other (P < 0.05) are indicated with different superscripts.

**Variables**	**Community Group 1 (n=10)** *Cleistogenes squarrosa/Carex duriuscula/ Allium polyrrhizum/ Artemisia frigida*	**Community Group 2 (n=9)** *Stipa krylovii*	**Community Group 3 (n=3)** *Agropyron cristatum/ Artemisia adamii/ Chenopodium album*	**Community Group 4 (n=3)** *Stipa gobica/ Kochia prostrata*	**Community Group 5 (n=2)** *Elymus chinensis*
Grass cover (%)	55.6±4.4	74.6±3.4	70.1±6.1	59.1±2.7	54.4±3.2
Forb cover (%)	10.2±3.9	10.51.6	5.9±2.1	9.2±1.6	1.4±0.2
Sedge cover (%)	10.8±3.7	0.8±0.2	3.6±2.4	0.1±0.1	4.2±3.4
Shrub cover (%)	2.5±0.4	0.9±0.4	2±0.2	0.9±0.3	1.4±0.6
Subshrub cover (%)	10.9±3.1	3.6±1.4	8.3±5.0	5.6±2.2	18±1.6
Palatable plant cover (%)	84.3±2.4	88.3±2.2	85.1±2.5	74.5±3.3	78.6±1.8
Unpalatable plant cover (%)	5.7±2.3	2.2±0.9	4.8±2.8	0.4±0.2	0.8±0.4
Annual plant cover (%)	0.72±0.3	0.8±0.6	1.7±1.2	0	0.2±0.2
Perennial plant cover (%)	89.3±1.9	89.7±2.3	88.1±1.2	74.9±3.1	79.2±2.4
Total foliar cover (%)	90±2	90.5±2.4	90.5±2.4	74.9±3.1	79.4±2.2
Grass biomass (g/m^2)	51.1±8.5	90.5±12.2	92.9±28.3	44.1±4.6	51.6±8.4
Forb biomass (g/m^2)	19.3±7.3	15.6±4.6	5.0±3.0	18.7±2	5.9±2.9
Sedge biomass (g/m^2)	9.1±5.6	0.4±0.2	4.7±4.5	0.3±0.3	0.1±0.1
Shrub biomass (g/m^2)	25.7±8.9	17.6±6.9	23.1±9.3	25.5±11.9	41.1±16.5

Total green biomass (g/m^2)	105.1±7.4	124±9.6	125.7±21.8	88.5±11	98.7±5.1
Litter biomass (g/cm^2)	20.2±7.1^{a}	35.6±5.2^{ab}	41±18.8^{ab}	23.8±6.9^{a}	79.6±12^{b}
Species richness (count)	28.3±2.8^{a}	27.2±3.6^{a}	26±7^{a}	25.7±2.3^{a}	20.5±1.5^{a}
Mean growing season precipitation (mm)	278.7±3.7	267.8±5.4	265.9±7.9	250.4±5.5	260.0±0
Mean annual precipitation (mm)	202.9±3	212.4±4.7	209.0±9.1	210.8±8.2	229.6±0
Mean annual temperature (^{o}C)	0.2±0.1	0.6±0.07	0.6±0.2	0.5±0.1	0.1±0
Aspect	128.3±18.9	164.4±13.9	134.7±10.1	154.0±18.3	188.5±8.5
Elevation (m)	1395.9±46.7	1254.9±34.2	1259.7±72.8	1244.3±34.6	1293.0±10
Slope (degrees)	7.5±1.4	6.0±1.2	10.0±3.1	6.7±1.2	5.5±0.5
