# Supplementary material for: Opportunities to integrate herders’ indicators into formal rangeland monitoring: an example from Mongolia
Source: Ecol Appl. 2019 May 17;29(5):e01899. doi: 10.1002/eap.1899 (PMC6851969; doi:10.1002/eap.1899)
Supplement: Supplementary file 7 [file EAP-29-na-s007.pdf]

Chantsallkham Jamsranjav, María E. Fernández-Giménez, Robin S. Reid, and B. Adya. 2019. Opportunities to integrate herders' indicators into formal rangeland monitoring: An example from Mongolia. *Ecological Applications.*

APPROVED

APPENDIX S7.

Table S1. Summary of selected vegetation, climate and environmental characteristics of community types in the desert steppe (DS) ecological zone. Values are means ± standard error (SE). Community groups were compared for differences in plant functional type cover and biomass, litter biomass and species richness using ANOVA and a Tukey-adjusted multiple comparison test. Groups that differed from each other ($P < 0.05$) are indicated with different superscripts.

**Variables**	**Community Group 1 (n=15)** *Allium mongolicum/ Salsola collina*	**Community Group 2 (n=4)** *Allium polyrrhizum/ Caragana stenophylla*	**Community Group 3 (n=5)** *Stipa gobica/ Allium mongolicum*	**Community Group 4 (n=3)** *Eragrostis minor/ Dontostemon integrifolius*
Grass cover (%)	7.3±1.9	9.0±2.3	22.3±2.5	13.7±2.5
Forb cover (%)	8.5±1.5	16.3±1.8	3.6±0.8	20.9±1.5
Sedge cover (%)	0.3±0.1	0.6±0.4	0.1±0.1	0.4±0
Shrub cover (%)	0.6±0.3	0.8±0.4	0.2±0.2	0.4±0.2
Subshrub cover (%)	0.9±0.2	0.3±0.2	0.6±0.3	1.7±0.5
Palatable plant cover (%)	15.6±2.1^{a}	25.6±2.2^{ab}	26±2.8^{b}	23.9±1.5^{ab}
Unpalatable plant cover (%)	2±0.6^{a}	1.4±0.9^{a}	0.8±0.5^{a}	13.3±3.9^{b}
Annual plant cover (%)	3.6±0.9	0.8±0.5	0.9±0.5	23.9±5.9
Perennial plant cover (%)	14.0±1.7	26.2±2.3	25.9±2.8	13.3±3.2
Total foliar cover (%)	17.6±2.1	27.0±2.6	26.8±3	37.2±3
Grass biomass (g/m^2)	2.5±0.7^{a}	4.7±1^{ab}	8.3±1.5^{b}	4.8±0.5^{ab}
Forb biomass (g/m^2)	4.6±0.8	9.3±1.6	2.0±0.4	6.4±1.4
Shrub biomass (g/m^2)	1.7±0.6	0.5±0.3	1.3±0.4	1.1±0.8
Sedge biomass (g/m^2)	0.2±0.08	0.3±0.1	0.02±0.02	0.2±0.09
Total green biomass (g/m^2)	9.0±1	14.8±1.7	11.6±2	12.5±.4
Litter biomass (g/m^2)	1.5±0.5	0.6±0.5	0.8±0.4	0.4±0.4

Species richness (count)	18.3±1.5[a]	22.3±3.2[a]	19.8±2.7[a]	26.7±1.2[a]
Mean growing season precipitation (mm)	78.6±3.7	94.4±4.8	88.9±4.2	99.2±0
Mean annual precipitation (mm)	123.4±2.2	130.9±0.3	131.7±0.2	131.2±0
Mean annual temperature (ºC)	3.0±0.1	2.4±0.2	2.6±0.02	2.9±0
Aspect	224.5±27	188.3±19.2	150.8±25.7	115.3±8.3
Elevation (m)	1189.1±11.7	1185.8±23.3	1200.4±45.1	1076.0±2.3
Slope (degrees)	2.0±0.2	2.0±0	1.0±0.3	1.7±0.3
